# A Novel Bio-Inspired Multi-Objective Enhanced Honey Formation Optimization Algorithm for Power Systems with Stochastic Renewable Resources

**DOI:** 10.3390/biomimetics11080539

**Published:** 2026-08-03

**Authors:** Mehmet Kaya, Hakan Işıker, Kadir Abacı

**Affiliations:** Electrical & Electronics Engineering Department, Faculty of Engineering, Mersin University, P.O. Box 33343 Mersin, Turkey; mehmet062@gmail.com (M.K.); kabaci@mersin.edu.tr (K.A.)

**Keywords:** Honey Formation Optimization, MO-HFO, MO-EHFO, multi-objective optimal power flow, economic–environmental and technical problem, renewable energy sources

## Abstract

The increasing prevalence of renewable energy sources (RESs), along with the uncertainty in their generation characteristics and conflicting economic, environmental, and technical objectives, has significantly increased the complexity of Economic–Environmental–Technical Dispatch (EETD) problems. Although numerous studies have been proposed in the literature for optimal power flow (OPF) and Economic Emission Dispatch (EED), comprehensive multi-objective EETD studies that account for stochastic renewable generation remain limited. Many existing Pareto-based approaches still exhibit insufficient convergence, poor solution diversity, and low-quality compromise solutions. To address these limitations, this paper proposes the Multi-Objective Enhanced Honey Formation Optimization (MO-EHFO) algorithm for EETD with integrated renewable energy. While preserving the five-phase basic structure of the original HFO, the MO-EHFO algorithm integrates archive-based, chaotic, and dynamic selective improvements for each phase to optimize population diversity, search capability, and convergence performance, using a Pareto-consistent approach. Evaluations conducted on a modified IEEE 30-bus system across nine case studies, each involving two, three, or four objectives, validate the exceptional performance of MO-EHFO. Additionally, the robustness of the proposed algorithm was tested using 25 simplified renewable energy-based scenarios representing specific times of the year. Simulation results show that while MO-EHFO satisfies all operational and safety constraints, it consistently produces superior Pareto fronts and high-quality compromise solutions compared to MO-HFO, NSGA-II, and the current literature. In conclusion, this study demonstrates that MO-EHFO is a reliable decision-support tool that provides users with the optimal balance between practicality, cost, and technical safety in complex and uncertain power system problems.

## 1. Introduction

The optimal power flow (OPF) problem, first introduced to the literature by Dommel and Tinney [1], has been a subject of extensive study. The primary objective of OPF is to optimize a specific objective function whilst satisfying physical, operational and security constraints [2].

Initially, the OPF was solved using classical quantitative optimization methods [3,4,5,6]; in subsequent years, with the widespread adoption of metaheuristic algorithms, it was solved using early versions of these methods [7,8,9,10] and improved versions [11,12]. Subsequently, several novel and effective methods such as biogeography-based optimization (BBO) [13], artificial bee colony algorithm (ABC) [14], gravitational search algorithm (GSA) [15], teaching–learning-based optimization technique (TLBO) [16], and differential search algorithm [17] were introduced in the literature. More recently, a developed particle swarm optimization method was proposed to solve the optimal power flow problem on the IEEE 30-bus system, minimizing generation fuel cost while satisfying all operational constraints [18]. Although most of these studies were originally designed to solve single-objective problems, they can also optimize multi-objective functions using the classical weighting method. In single-objective OPF problems, the solution point optimizes the objective function while other objectives are pushed to upper bounds. There is a growing need for multi-objective optimal power flow (MO-OPF) studies that offer more realistic and sustainable operational solutions that balance economic, environmental, technical, and security objectives simultaneously, rather than single-objective (OPF) approaches. Traditional optimization procedures yield a single solution point, whereas MO methods provide a set of optimal solutions, known as the Pareto set. Increases in fuel costs and environmental concerns have led to the development of multi-objective evolutionary algorithms (MOEAs) to address the multi-objective OPF problem, which simultaneously considers objectives such as minimizing active and reactive losses, voltage deviation, voltage stability index, and emissions [2]. In addressing this aspect, the authors of [19] employed the shuffle frog leaping algorithm (SFLA) to tackle the multi-objective OPF problem, with a particular emphasis on emissions. In [20], the Pareto front was determined using Fast Elitist Nondominated Sorting and Crowding Distance (NSMOGSA) methods, and the continuous control variables in the OPF problem were optimally adjusted using an external solution archive. Another approach that was introduced was the Multi-Objective Evolutionary Algorithm-Based Decomposition (MOEA/D), using a modified Tchebycheff decomposition method to obtain uniformly distributed Pareto-optimal solutions on each objective space. In addition to these advancements, various Pareto concept-based meta-heuristic techniques have been implemented, including the multi-objective evolutionary algorithm based on decomposition superiority of feasible solutions (MOEA/D-SF) [21].

The swift emergence of renewable energy sources (RESs) within contemporary energy systems has underscored the imperative for the concurrent optimization of energy production, aiming to attain economic, environmental, and security objectives [22]. In parallel, multi-objective optimization has also been applied to renewable-rich settings beyond conventional dispatch; for example, a comparative multi-objective analysis of peer-to-peer energy trading in a rooftop-photovoltaic residential community jointly optimized renewable-energy utilization and grid-cost objectives [23]. However, the inherent uncertainty associated with RESs introduces a degree of complexity into the OPF problem [24], thus necessitating the adoption of various optimization approaches. In the context of renewable, complex, and non-linear OPF problems where objective functions are being minimized simultaneously, the Pareto approach has been shown to produce more reliable results than the weighted sum. Pareto-based multi-objective optimization is a methodology that has been demonstrated to reveal the true balance between conflicting objectives in power systems.

In light of the mounting environmental concerns, numerous studies have explored the subjects of Economic Emission Dispatch (EED) and Dynamic Economic Emission Dispatch (DEED) in conjunction with the conventional OPF methodology. In [25], the authors proposed the MOEA/D method for a wind–thermal energy system to simultaneously optimize both cost and emissions. Moreover, the dynamic economic dispatch optimization problem encompassed the consideration of the generator’s emissions and valve point loading effects [26]. Extending this line of research toward learning-based dispatch, Keskin [27] introduced a Two-Stage Proximal Policy Optimization–Reinforcement Learning Marine Predators Algorithm (PPO-RLMPA) framework that couples a constraint-aware PPO agent with a Marine Predators Algorithm adaptively tuned by a Deep Q-Network; across ten- and twenty-unit benchmark systems integrating wind, photovoltaic, and pumped-storage hydro resources, the framework achieved cost reductions of approximately 1.1% and 4.4% over the Chaotic Fast Convergence Evolutionary Programming (CFCEP) baseline with zero constraint violations in all 30 independent runs. More recently, Zhang and Li [28] proposed an Improved Pelican Optimization Algorithm (IPOA) that integrates a vertical crossover operator and a dimensional variation strategy into the standard Pelican Optimization Algorithm; tested on Economic Load Dispatch (ELD) benchmark systems with up to 80 generating units, IPOA reduced coal consumption by up to 3.67% relative to the baseline POA, confirming that behavior-inspired search enhancements deliver meaningful efficiency gains in large-scale power generation scheduling. The growing penetration of RESs, together with increasingly stringent environmental regulations, has significantly heightened the importance of EED formulations, where fuel cost and emission are optimized simultaneously under system operating constraints.

Although the EED problem yields positive economic and environmental results, neglecting the technical operation of power systems leads to issues such as increased power loss, voltage deviation, and reduced voltage stability. Consequently, Economic–Environmental–Technical Dispatch (EETD) has emerged as a more comprehensive optimization framework by incorporating technical performance indices into the conventional EED formulation. EETD directly addresses the coordinated dispatch of generating units while simultaneously considering economic efficiency, environmental sustainability, and technical security under stochastic renewable generation. This capability makes EETD particularly suitable for modern renewable-integrated power systems, where the intermittent nature of wind, solar, and hydro resources continuously changes the generation mix and significantly complicates the dispatch process.

EETD research has gained momentum recently. For instance, the study in [29] formulates the OPF problem as a Many-Objective OPF (Ma-OPF) problem, aiming to minimize multiple objective functions—including total fuel cost (TFC), total emissions (TE), voltage deviation (VD), power loss (PL), and L-index under complex constraints. Furthermore, Ref. [29] utilizes a weighted sum strategy based on the Analytic Hierarchy Process (AHP) to convert the multi-objective problem into a normalized single-objective one. In [30], a multi-objective EETD model that simultaneously accounts for economic, environmental, and technical criteria was developed for IEEE 30- and 57-bus systems with high renewable-energy penetration, employing the Coronavirus Herd Immunity Optimizer (CHIO), the Salp Swarm Algorithm (SSA), and the Ant Lion Optimizer (ALO); the proposed CHIO outperformed the competing methods. Finally, the equilibrium optimization (EO) algorithm and its multi-objective version, recently published for systems including wind, solar, and combined solar and hydro power plants, have been proposed to address both single and MO-OPF problems [31]. In a complementary study on combined heat and power dispatch, Nazari and Abdi [32] proposed the Imperialist Competitive Harris Hawks Optimization (ICHHO) algorithm—a bio-inspired hybrid merging the global exploration of the Imperialist Competitive Algorithm with the rapid exploitation of the Harris Hawks Optimizer—to address the Combined Heat and Power Economic Dispatch (CHPED) problem; validated on medium- to very-large-scale systems of 24 to 96 units, ICHHO reduced minimum operation costs by at least 0.19–0.34% compared to leading methods in the literature.

Despite its practical importance, comprehensive multi-objective EETD studies that account for stochastic renewable generation remain relatively limited in the existing literature. Nevertheless, the existing EETD literature still exhibits several important limitations. First, the number of studies addressing stochastic renewable-integrated EETD remains relatively small compared with the extensive body of research on OPF and EED problems. Second, although Pareto-based optimization has become the dominant approach for solving multi-objective dispatch problems, many existing methods fail to accurately approximate the true Pareto front, often producing poorly distributed compromise solutions and exhibiting limited convergence. Moreover, some reported approaches violate operational constraints or produce solution sets that are difficult to compare fairly due to inconsistent constraint handling and inadequate preservation of diversity. These shortcomings reduce both the physical reliability of the obtained operating points and the transparency of performance comparisons among competing optimization methods.

Motivated by these research gaps, this paper first employs the Honey Formation Optimization (HFO) algorithm in a multi-objective framework. Subsequently, it develops a Multi-Objective Enhanced Honey Formation Optimization (MO-EHFO) algorithm for stochastic, renewable-integrated EETD problems. By incorporating enhanced archive management, adaptive search mechanisms, improved diversity preservation, and Pareto-guided search strategies, the proposed approach aims to generate well-distributed, physically feasible Pareto-optimal solutions while improving convergence accuracy, compromise quality, and constraint satisfaction under highly conflicting operating objectives.

Within this search for better alternatives, the Honey Formation Optimization (HFO) algorithm was recently introduced for design problems [33] and later adapted to numerical function optimization [34] and various engineering applications such as solar energy systems [35], food processing [36,37], and water and climate change [38]. Also, HFO has been successfully adapted to the OPF problem [39]. However, since the traditional HFO was originally designed for single-objective functions, its direct adaptation to a multi-objective framework (MO-HFO) demonstrated insufficient performance. This shortcoming necessitated the development of a more robust approach, which resulted in the development of EHFO. The latter optimizes the core HFO component within the MO framework, thus enabling superior convergence to the Pareto front and facilitating the solution of MO-OPF problems. This article offers solutions to improve economic, environmental, and technical performance using the MO-EHFO method proposed here, while addressing uncertainties in wind, solar, and hydroelectric resources, without violating system constraints.

To rigorously validate the superiority and validity of the proposed method, comprehensive case studies were conducted using MO-EHFO, MO-HFO, and the Non-dominated Sorting Genetic Algorithm II (NSGA-II). NSGA-II was specifically selected for these performance evaluations because it is a highly competitive, state-of-the-art benchmark algorithm that is widely accepted as a reference standard in the field of multi-objective optimization problems [40]. The comparative case studies demonstrate that the MO-EHFO algorithm provides highly competitive Pareto front optimization when evaluated against these robust baseline algorithms.

The main contributions of this paper are summarized as follows:

• A stochastic renewable-integrated multi-objective EETD problem is solved for the first time using the proposed MO-EHFO algorithm.
• MO-EHFO produces superior Pareto fronts and higher-quality compromise solutions than existing methods while satisfying all operational and security constraints.
• A comprehensive scenario-based validation demonstrates the robustness and stability of MO-EHFO under different seasonal renewable generation conditions.
• The proposed MO-EHFO integrates chaotic initialization, adaptive search, personal Pareto archives, and archive-guided leader selection to improve convergence and solution diversity.
• The proposed framework provides an effective decision-support tool for balancing economic, environmental, and technical objectives in modern renewable-integrated power systems.

## 2. Mathematical Formulation of MO-OPF

With the integration of RESs such as wind, solar, and hydro, the MO-OPF problem has evolved into a complex optimization framework characterized by nonlinear power flow equations, system operating constraints, and uncertainties. In this section, the mathematical formulation of the MO-OPF problem is presented, and the objective functions, power flow equations, and system operating constraints are defined in detail.

(1) $$\begin{array}{c} \underset{x,u}{min}\;F\left(x,u\right)=\left[f_{1}\left(x,u\right),f_{2}\left(x,u\right),\ldots ,f_{M}\left(x,u\right)\right]\\ subject\;to:\\ g(x,u)=0,\;h(x,u)\leq 0 \end{array}$$

where $F\left(x,u\right)$ represents the vector of multi-objective functions, while g(x,u) and h(x,u) denote the power flow equality constraints and the system operating inequality constraints, respectively.

(2) $$x=\left[P_{Gslack}V_{L}{\;Q}_{G}S_{TL}\right],\mathrm{and}\;u=\left[\begin{array}{ll} P_{G} & V_{G} \end{array}\right]$$

where u represents the vector of control variables, comprising the active power generation $P_{G}$ and generator voltages $V_{G}$. On the other hand, x denotes the vector of state variables, which includes the active power generation of the slack bus $P_{Gslack}$, load bus voltages $V_{L}$, reactive power generation outputs $Q_{G}$, and transmission line loadings $S_{TL}$.

### 2.1. Constraints of the Power System

#### 2.1.1. Equality Constraints

The equality constraints represent the nodal power balance equations, ensuring that the net scheduled power injection at bus k matches the calculated power flow. In this formulation,

(3) $$\begin{array}{rr} P_{k}^{G}-P_{k}^{D} & =\sum_{l=1}^{N}\;\;V_{k}V_{l}\left(G_{kl}cos\left(\theta_{k}-\theta_{l}\right)+B_{kl}sin\left(\theta_{k}-\theta_{l}\right)\right)\\ Q_{k}^{G}-Q_{k}^{D} & =\sum_{l=1}^{N}\;\;V_{k}V_{l}\left(G_{kl}sin\left(\theta_{k}-\theta_{l}\right)-B_{kl}cos\left(\theta_{k}-\theta_{l}\right)\right) \end{array}$$

where $P_{k}^{G}$ and $Q_{k}^{G}$ are the active and reactive power generated, while $P_{k}^{D}$ and $Q_{k}^{D}$ are the active and reactive load demands at thebus. The system state is defined by the voltage magnitudes V and angles θ for buses k and l. Furthermore, $G_{kl}$ and $B_{kl}$ represent the mutual conductance and susceptance values derived from the system admittance matrix, where N is the total number of buses.

#### 2.1.2. Inequality Constraints

The active and reactive power generation limits for each generation unit are defined as follows:

(4) $$\begin{array}{rr} \begin{array}{c} P_{k,min}^{G}\leq P_{k}^{G}\leq P_{k,max}^{G}\\ Q_{k,min}^{G}\leq Q_{k}^{G}\leq Q_{k,max}^{G}\\ V_{k,min}^{G}\leq V_{Gk}\leq V_{k,max}^{G} \end{array} & ,\;\;k=1,\ldots \ldots ,N_{g} \end{array}$$

where $P_{k,min}^{G}$ ($P_{k,max}^{G}$), $Q_{k,min}^{G}$ ($Q_{k,max}^{G}$), and $V_{k,min}^{G}$ ($V_{k,max}^{G}$) denote the minimum (maximum) limits for active power generation, reactive power generation, and generator bus voltage magnitude at bus k, respectively, while $N_{g}$ represents the total number of generation units.

#### 2.1.3. Security Constraints

To guarantee the security and reliability of the power system, the state variables must be maintained within strictly defined technical limits. These security constraints encompass the voltage magnitude limits for both generator and load buses, as well as the thermal capacity limits of transmission lines. The voltage constraints for load buses are formulated as follows:

(5) $$V_{L,m}^{min}\leq V_{L,m}\leq V_{L,m}^{max},\;m=1,\ldots ,N_{L}$$

Furthermore, the apparent power flow through each transmission line must not exceed its maximum thermal rating:

(6) $$S_{l}\leq S_{l}^{max},\;\;l=1,\ldots ,N_{line}$$

where $N_{line}$ is the total number of transmission lines.

##### Prohibited Operating Zones (POZs)

Physical limitations in the mechanical components of thermal power plants, such as steam valve vibrations or shaft instabilities, may create discontinuous operating regions known as prohibited operating zones (POZs). To prevent mechanical damage, the power output of the k-th thermal unit must avoid these specific zones. Consequently, the operating region of a unit with $N_{poz}$ number of prohibited zones is defined as a set of disjoint feasible sub-regions:

(7) $$P_{TGk}^{\min\text{\_}\mathrm{poz},j}<{POZ}_{TGk}^{j}<P_{TGk}^{\max\text{\_}\mathrm{poz},j},\;\;j=1,\ldots ,N_{poz}$$

where $P_{TGk}^{\min\text{\_}\mathrm{poz},j}$ and $P_{TGk}^{\max\text{\_}\mathrm{poz},j}$ represent the lower and upper bounds of the j-th prohibited operating zone for the k-th thermal unit, respectively [41].

### 2.2. Modeling Stochastic RESs

The integration of wind, solar, and hydroelectric energy sources into power systems necessitates the use of stochastic modeling approaches in OPF studies due to the inherent uncertainties in their generation. In this context, the available power outputs for hydro, solar, and wind sources are calculated using Gumbel, lognormal, and Weibull probability density functions (PDFs), respectively. Accordingly, a detailed explanation of the probabilistic modeling of these RESs is provided in this section.

#### 2.2.1. Stochastic Modeling in Wind Energy

**Figure 1:**
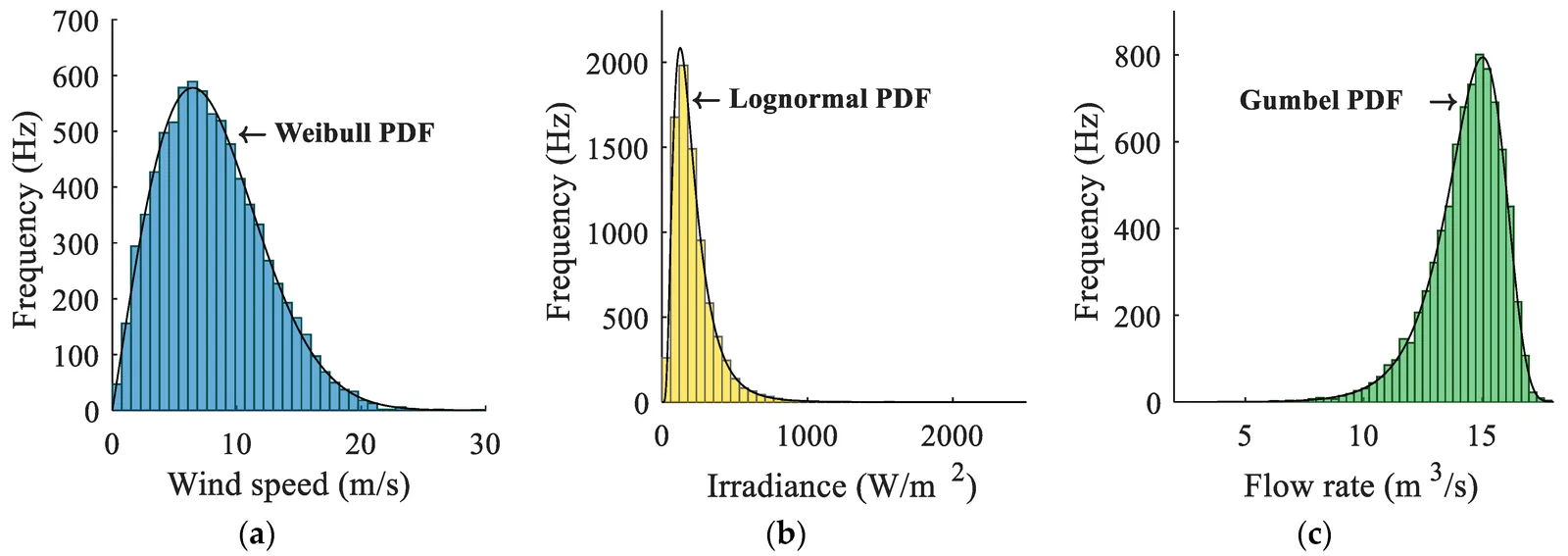
RESs Weibull, lognormal, and Gumbel PDF models for (**a**) wind, (**b**) solar, and (**c**) hydro.

The stochastic behavior of wind speed is a critical factor in power generation and is mathematically modeled using the Weibull PDF as depicted in Figure 1a. The probability of a specific wind speed, v (m/s), is expressed as follows [42]:

(8) $$f_{w}\left(v\right)=\left(\frac{k}{c}\right).{\left(\frac{v}{c}\right)}^{\left(k-1\right)}.e^{-{\left(v/c\right)}^{k}},\;\;\;0<v<\infty$$

where k and c represent the dimensionless shape parameter and the scale parameter of the Weibull distribution, respectively [43].

To determine the active power output of a wind turbine based on the stochastic wind speed, the turbine’s specific power curve characteristics are utilized. The transfer function relating wind speed to generated power $P_{W}$ is defined as:

(9) $$\begin{array}{ccc} P_{W\left(v\right)} & = & \left\{\begin{array}{cc} 0 & \;\;v<v_{in}\;or\;v>v_{out}\\ P_{Wr}\left(\frac{v-v_{in}}{v_{r}-v_{in}}\right) & v_{in}\leq v\leq v_{r}\\ P_{Wr} & v_{r}<v\leq v_{out} \end{array}\right. \end{array}$$

Here, $v_{in}$, $v_{out}$, and $v_{r}$ denote the cut-in, cut-out, and rated wind speeds of the turbine, respectively, while $P_{Wr}$ indicates the rated power output of the wind generation unit.

#### 2.2.2. Stochastic Modeling in Solar Power

The power output of solar photovoltaic (PV) systems is directly correlated with solar irradiance levels. The uncertainty associated with solar irradiance $G_{s}$ is typically characterized by a lognormal PDF as shown in Figure 1b. The probability distribution function for solar irradiance, defined by a mean $\mu_{s}$ and standard deviation $\sigma_{s}$, is formulated as [43]:

(10) $$f_{G}\left(G_{s}\right)=\frac{1}{{G_{s}\sigma }_{s}\sqrt{2\pi }}e^{\left[-\frac{{\left(\ln G_{s}-\mu_{s}\right)}^{2}}{2\sigma_{s}^{2}}\right]},\;\;G_{s}>0$$

Consequently, the active power generated by the PV source $P_{s}$ as a function of solar irradiance is calculated using the following piecewise function:

(11) $$\begin{array}{cccc} P_{s\left(G_{s}\right)} & = & \left\{\begin{array}{c} P_{sr}\left(\frac{G_{s}^{2}}{G_{std}.R_{C}}\right),\;\\ P_{sr}\left(\frac{G_{s}}{G_{std}}\right), \end{array}\right. & \begin{array}{c} \;0<\;G_{s}<\;X_{C}\\ G_{s}\geq R_{C} \end{array} \end{array}$$

where $P_{sr}$ represents the rated power of the solar PV unit, $G_{std}$ is the standard solar irradiance in the standard environment, and $R_{c}$ denotes the specific irradiance point.

#### 2.2.3. Stochastic Modeling in Hydro Power

The potential power output of a run-of-river hydroelectric power plant depends heavily on the river flow rate [44]. The stochastic nature of the annual peak flow discharge Q is modeled using the Gumbel PDF as seen in Figure 1c. The PDF for the river discharge is given by:

(12) $$f_{Q}\left(Q\right)=\frac{1}{\beta }\exp\left(-\frac{Q-\mu_{H}}{\beta }-\exp\left(-\frac{Q-\mu_{H}}{\beta }\right)\right)$$

where μ and β represent the location and scale parameters of the Gumbel distribution, respectively.

Based on the stochastic river discharge Q, the generated power output of the hydro unit $P_{H}$ is calculated using the fundamental hydroelectric power equation:

(13) $$P_{H}\left(Q\right)=\eta \cdot \rho \cdot g\cdot Q\cdot H_{net}$$

where η is the overall efficiency of the turbine-generator set as 0.85, ρ is the water density (1000 kg/m^3^), g is the gravitational acceleration (9.81 m/s^2^), and $H_{net}$ denotes the net hydraulic head available at the plant.

All other parameters related to RESs integrated into the modified IEEE 30-bus test system are provided in Table 1.

### 2.3. Mathematical Models for Generation Costs

The objective function of the proposed MO-OPF problem includes the minimization of total generation costs. These costs comprise the fuel costs of thermal units and the direct, reserve, and penalty costs associated with RESs.

#### 2.3.1. Fuel Cost of Thermal Unit

The operational cost of thermal generators is primarily derived from fossil fuel consumption. While traditionally represented by a quadratic function, accurate modeling requires the inclusion of the valve-point loading effect, which introduces a rippling behavior in the cost curve [41]. Therefore, the total fuel valve cost ${(C}_{Tv})$ is formulated as the superposition of a quadratic polynomial and a rectified sinusoidal term:

(14) $$C_{Tv}\left(P_{T}\right)=\sum_{k=1}^{N_{T}}\left(a_{k}+b_{k}P_{T,k}+c_{k}P_{T,k}^{2}+\left|d_{k}\sin\left(e_{k}\left(P_{T,k}^{min}-P_{T,k}\right)\right)\right|\right)$$

where $P_{T,k}$ represents the active power output of the k-th thermal unit. The coefficients $a_{k}$, $b_{k}$, $c_{k}$ denote the fuel cost coefficients, while $d_{k}$ and $e_{k}$ are the coefficients modeling the valve-point loading effect. $N_{T}$ indicates the total number of thermal generators.

#### 2.3.2. Cost of Wind, Solar and Hydro Power

Due to the stochastic nature of RESs, their cost functions are evaluated in three distinct components:

The direct cost for the scheduled power;
The reserve cost for power underestimation (deficit);
The penalty cost for power overestimation (surplus).

##### Direct Cost

It is assumed that the system operator enters into a contractual agreement to purchase a specific amount of scheduled power from private renewable energy providers. The direct cost represents the payment for this scheduled power $\left(P_{sch}\right)$ and is defined linearly for RESs as follows:

(15) $$C_{d,W}=\sum_{i=1}^{N_{W}}K_{d,W,i}\cdot P_{W,sch,i}$$

(16) $$C_{d,S}=\sum_{j=1}^{N_{S}}K_{d,S,j}\cdot P_{S,sch,j}$$

(17) $$C_{d,H}=\sum_{m=1}^{N_{H}}K_{d,H,m}\cdot P_{H,sch,m}$$

where $K_{d}$ represents the direct cost coefficient for the respective energy source, which is 1.7 for wind turbines, 1.6 for solar, and 1.5 for hydro [31].

##### Reserve Cost

If the actual power generation falls below the scheduled amount, the system operator must activate spinning reserves to maintain the load–generation balance. This incurs a reserve cost, which is calculated based on the expected power deficit:

(18) $$C_{r,W}=\sum_{i=1}^{N_{W}}K_{r,W,i}\int_{0}^{P_{W,sch,i}}\left(P_{W,sch,i}-p\right)f_{W}\left(p\right)dp$$

(19) $$C_{r,S}=\sum_{j=1}^{N_{S}}K_{r,S,j}\int_{0}^{P_{S,sch,j}}\left(P_{S,sch,j}-p\right)f_{S}\left(p\right)dp$$

(20) $$C_{r,H}=\sum_{m=1}^{N_{H}}K_{r,H,m}\int_{0}^{P_{H,sch,m}}\left(P_{H,sch,m}-p\right)f_{H}\left(p\right)dp$$

where $K_{r}$ represents the reserve cost coefficient for the relevant energy source; this coefficient has been set to 3 for all RESs [45].

##### Penalty Cost

Conversely, if the actual available power exceeds the scheduled amount, the surplus power may be curtailed if it cannot be absorbed by the grid. This scenario results in a penalty cost for the unutilized available energy:

(21) $$C_{p,W}=\sum_{i=1}^{N_{W}}K_{p,W,i}\int_{P_{W,sch,i}}^{P_{W,r}}\left(p-P_{W,sch,i}\right)f_{W}\left(p\right)dp$$

(22) $$C_{p,S}=\sum_{j=1}^{N_{S}}K_{p,S,j}\int_{P_{S,sch,j}}^{P_{S,r}}\left(p-P_{S,sch,j}\right)f_{S}\left(p\right)dp$$

(23) $$C_{p,H}=\sum_{m=1}^{N_{H}}K_{p,H,m}\int_{P_{H,sch,m}}^{P_{H,r}}\left(p-P_{H,sch,m}\right)f_{H}\left(p\right)dp$$

where $K_{p}$ represents the penalty cost coefficient for the relevant energy source, this coefficient has been set to 1.4 for all RESs, and $P_{r}$ indicates the rated capacity of the respective generation unit [45].

## 3. Test System

In this study, the MO-OPF problem is solved for the modified IEEE 30-bus test system integrated with RESs by simultaneously minimizing five conflicting objective functions, the formulations of which are presented below.

To guarantee that the power system operates strictly within its security and operational limits, a penalty function technique was employed. This approach ensures that all state and control variables remain within their specified feasible ranges.

The proposed method is evaluated on a modified IEEE 30-bus test system. In this configuration, thermal generation units are located at buses 1, 2, and 8. Regarding renewable energy integration, a wind power unit is installed at bus 5, a standalone solar PV unit at bus 11, and a small hydro plant integrated with a hybrid PV system at bus 13. The fundamental parameters of the modified test system are summarized in Table 2, while the specific emission and fuel cost coefficients are adopted from Ref. [45].

### 3.1. Total Generation Cost $\left(F_{CT}\right)$

The primary objective function, $F_{1}$, represents the aggregate operational cost of the power system. This function is constructed by superimposing the fuel costs of thermal units and the probabilistic costs (direct, reserve, and penalty) associated with RESs, including wind, solar, and hydro units, and its equations are given in (14) to (23).

(24) $$F_{CT}\left(x,u\right)=\sum_{k=1}^{N_{T}}C_{Tv,k}\left(P_{T,k}\right)+\sum_{x\in \{W,S,H\}}\sum_{i=1}^{N_{x}}\left(C_{d,x,i}+C_{r,x,i}+C_{p,x,i}\right)$$

where $N_{T}$, $N_{W}$, $N_{S}$, and $N_{H}$ represent the number of thermal units, total number of wind, solar, and hydro generation units, respectively.

### 3.2. Environmental Emissions $\left(F_{E}\right)$

To mitigate the environmental footprint of fossil-fuel-based generation, the second objective function minimizes the emission of harmful pollutants such as $NO_{x}$ and $SO_{x}$ from thermal power plants. The emission characteristics are modeled using a combination of a quadratic polynomial and an exponential term:

(25) $$F_{E}\left(x,u\right)=\sum_{k=1}^{N_{T}}\left[\left(\alpha_{k}+\beta_{k}P_{T,k}+\gamma_{k}P_{T,k}^{2}\right)+\omega_{k}\exp\left(\mu_{k}P_{T,k}\right)\right]$$

where $\alpha_{k}$, $\beta_{k}$, $\gamma_{k}$ , $\omega_{k}$ , and $\mu_{k}$ are the specific emission coefficients associated with the k-th thermal generation unit.

### 3.3. Total Active Power Loss $\left(F_{PL}\right)$

The third objective aims to enhance the transmission efficiency of the network by minimizing the total active power losses ($P_{loss}$) occurring across transmission lines. This objective is derived from the line conductance and the voltage phase angle differences:

(26) $$F_{PL}\left(x,u\right)=\sum_{l=1}^{N_{line}}G_{kl}\left[V_{k}^{2}+V_{l}^{2}-2V_{k}V_{l}\cos\left(\theta_{k}-\theta_{l}\right)\right]$$

### 3.4. Voltage Deviation $\left(F_{VD}\right)$

The fourth objective addresses the power quality and security of the system. Minimizing the voltage deviation (VD) ensures that the voltage profile at load buses remains close to the nominal reference value (1.0 p.u.), thereby improving system stability:

(27) $$F_{VD}\left(x,u\right)=\sum_{k=1}^{N_{L}}\left|V_{L,k}-1.0\right|$$

where $N_{L}$ represents the total number of load buses and $V_{L,k}$ denotes the voltage magnitude at the k-th load bus.

### 3.5. Voltage Stability Index $\left(F_{VSI}\right)$

Ensuring static voltage stability is paramount for the reliable operation of power systems, especially under heavy loading conditions. The L-index is widely utilized as a robust metric to evaluate the voltage stability margin of load buses and to detect the proximity of the system to voltage collapse.

The L-index ($L_{j}$) for any load bus j is mathematically formulated as follows [47]:

(28) $$L_{j}=\left|1-\sum_{i=1}^{N_{G}}F_{ji}\frac{V_{i}}{V_{j}}\right|,\;j=1,2,\ldots ,N_{L}$$

where $F_{ji}$ represents the elements of the complex matrix derived from the bus admittance matrix ($Y_{BUS}$).

(29) $$F_{ji}=-{\left[Y_{LL}\right]}^{-1}\left[Y_{LG}\right]$$

where $Y_{LL}$ and $Y_{LG}$ correspond to the sub-matrices of the $Y_{BUS}$ matrix, representing the load–load and load–generator admittances, respectively.

Consequently, the objective function for maximizing voltage stability is defined as the maximum L-index value observed in the system:

(30) $$F_{VSI}\left(x,u\right)=L_{max}=\max\left(L_{j}\right),\;\forall j\in \{1,\ldots ,N_{L}\}$$

## 4. Multi-Objective Honey Formation Optimization Algorithm

The HFO with Single Component (HFO-1), which models the honey production process of honey bees in nature, is a powerful metaheuristic algorithm developed for numerical function optimization [34]. The fundamental HFO-1 algorithm is designed to optimize problems over a single objective function. However, the necessity to simultaneously optimize multiple conflicting objectives inherent in real-world problems has mandated the evolution of this basic structure into a multi-objective framework. In this study, the fundamental HFO-1 architecture is first introduced, followed by a step-by-step detailing of its evolutionary transition into multi-objective variants MO-HFO and MO-EHFO.

### 4.1. HFO-1

The HFO-1 algorithm simulates the process from the initial formation of honey in the bee’s stomach to its maturation and saturation within the hive across five fundamental phases (initialization, honey formation, mixing, maturation, and saturation). The core skeleton of the algorithm and the locations of the mathematical equations utilized within this framework are presented in Algorithm 1 HFO-1.
**Algorithm 1. HFO-1 (Single-Objective Base Framework)**1-**//PHASE 1: INITIALIZATION**2-*Sources* ← initialize_sources()/**/**Positions and costs are calculated using Equations (31) and (32)3-initialize_variables()/**/**Parameters and timers are initialized using Equations (33)–(35)4-*Gbest* ← *Pbest* ← *Sources*[mx]/**/**Leaders are assigned with a random initial index, e.g., mx = 15-**repeat**6-**//PHASE 2: HONEY FORMATION**7-
**for each ***source* of workers/**/Worker bee phase**8-

*vi* ← local_search(*source*)/**/**Candidate solution is generated using Equations (36) and (37)9-

**if ***vi.CCost* < *source.CCost* **then** replace *source* with *vi*10-
*P* ← selection_probabilities(*Sources*)/**/**Roulette probabilities are calculated using Equations (38) and (39)11-
**for each ***onlooker*/**/**Onlooker bee phase12-

*source* ← select_by_roulette(*P*)13-

*vi* ← local_search(*source*)/**/**Candidate solution is generated using Equations (36) and (37)14-

**if ***vi.Cost* < *source.Cost* **then** replace *source* with *vi*15-**//PHASE 3: MIXING**16-
**if** mixing phase condition is met/**/**It is checked via Equation (40)17-

*Sources* ← mix honey-forms/**/**Crossover between Pbest and neighbors is performed using Equations (41) and (42)18-
ensure the *Pbest* form is not corrupted19-**//PHASE 4 & 5: MATURATION & SATURATION**20-
**if** maturation condition is met/**/**Homogeneity check under epsilon precision linked to Equation (34)21-

*Sources* ← initialize_sources()/**/**If the site is mature, transition to a new site (newSite = true)22-
**else if** saturation condition is met23-

*Sources*[*mx*] ← inject *Gbest*/**/**Gbest is saturated to accelerate the maturation of the new mixture24-
**if ***Gbest* is not improved compared to *pGbest* for one maturation period25-

change *mixSize* randomly/**/**Automatic parameter tuning is performed from the pool in Equation (33)26-**until** maximum iteration

The mathematical framework and functions that constitute the core of the HFO-1 algorithm are explained in sequence according to the relevant phases:

#### 4.1.1. HFO-1 Phase 1: Initialization

This phase is where candidate solutions in the search space and the global parameters governing the intra-hive dynamics are defined.

##### Source Initialization (Initialize_Sources())

Candidate solutions (sources) are initialized as a structure [Position, Cost, CCost] comprising the position, standard objective function cost, and component cost. The position vector of the $i^{th}$ solution, $x_{i}$, is generated using a uniform random distribution between the lower ($x^{min}$) and upper ($x^{max}$) bounds of the problem space according to (31).

(31) $$x_{i,d}=x_{d}^{min}+rand\cdot \left(x_{d}^{max}-x_{d}^{min}\right)$$

Here, d ∈ {1, 2, …, D} denotes the problem dimension being updated, D is the total number of dimensions, and $rand\in \left[0,1\right]$ represents a uniform random number. The component cost ($CCost_{x_{i}}$), calculated to guide the worker bees, is the difference between the standard cost of the current source ($Cost_{x_{i}}$) and the cost of the globally best solution found so far (Gbest). Initially, Gbest.Cost=0 is assumed.

(32) $$CCost_{x_{i}}=Cost_{x_{i}}-Gbest.Cost$$

##### Variable Initialization (Initialize_Variables())

The maximum dimension boundary array, which will constrain the mixing and search dimensions in subsequent phases, is initialized as mixSize=[D,D,D]. An index mx, utilized to track the population best of the previous iteration, is arbitrarily assigned (e.g., mx=1) to serve as a reference for the algorithm. The population best (Pbest) and Gbest are equated to this index and fed into the loop. To measure stagnation within the system, a variable pGbest is defined, representing the global best of the previous maturation period. A random dimension pool (randPool), to be employed for escaping local traps (tuning) during the saturation phase, is constructed via (33).

(33) $$randPool=\left\{\begin{array}{ll} {}[1,\lceil D/4\rceil ,D], & \mathrm{if}\;D>2\\ {}[D], & \mathrm{otherwise} \end{array}\right.$$

To prevent division by zero, a base precision is set to $precision=1e^{-3}$, and the tolerance limit (siteLim) to be used in floating-point comparisons is formulated by (34).

(34) $$siteLim=abs({log}_{10}(precision))+2$$

To establish the phase transition thresholds of the system, a mixing period factor (MF) is utilized, and all timers are configured as a unified interconnected system in (35).

(35) $$\begin{array}{c} \;mixPeriod=16\cdot MF\\ \;\;\;\;maturePeriod=4\cdot mixPeriod\\ \;\;\;\;sitePeriod=4\cdot maturePeriod\\ finalizationPeriod=4\cdot sitePeriod \end{array}$$

#### 4.1.2. HFO-1 Phase 2: Honey Formation

This is the phase where worker and onlooker bees continue honey production by performing local searches around their current sources.

##### Local Search (Local_Search())

To generate a new neighbor candidate solution $v_{i}$ around the current source $x_{i}$, the vectorial search equation in (36) is applied.

(36) $$v_{i,j}=x_{i,j}+q\cdot \left(x_{i,j}-x_{k,j}\right)$$

In this equation, $x_{k}\neq x_{i}$ is a distinct neighbor randomly selected from the population. j is a list of selected dimensions (indices) specifically chosen to update the candidate ($v_{i}$) in this phase. The quantity of these dimensions to be updated (ms) is determined using the maximum mixing bound via randi(mixSize[3]), and the search is executed solely over these selected j indices. The parameter q, which dictates the search step size, is a piecewise function dependent on the initial step size (qo) and a Boolean variable (randWalk) controlling the random walk permission in (37).

(37) $$q=\left\{\begin{array}{ll} \lceil 1/(4\cdot rand)\rceil , & \mathrm{if}\;randWalk\;\mathrm{is}\;\mathrm{true}\;\&\;rand<0.5\\ qo, & \mathrm{otherwise} \end{array}\right.$$

##### Selection Probabilities (Selection_Probabilities())

The probabilities ($p_{i}$) of onlooker bees migrating towards sources with lower costs are calculated using (38) and (39) based on the minimum cost (mincost) present in the population of size $N_{pop}$:

(38) $$fit_{x_{i}}=\frac{1}{Cost_{x_{i}}-mincost+precision}$$

(39) $$qp_{i}=\frac{fit_{x_{i}}}{\sum_{k=1}^{N_{pop}}fit_{x_{k}}}$$

#### 4.1.3. HFO-1 Phase 3: Mixing

This phase involves the bees sharing their forms within the hive among themselves or with the primal catalyzer, Pbest.

##### Mixing Procedure (Mixing_Procedure())

Based on the local stagnation counter of the source ($C_{i}$) and the global period counters (c1,c2), the formation level (Raw = 0, Semi-Mature = 1, Mature = 2) is determined by (40).

(40) $$formation\_level=\left\{\begin{array}{ll} 2, & \mathrm{if}\;c2>4\cdot mixPeriod\;\mathrm{and}\;C_{i}>4\cdot mixPeriod\\ 1, & \mathrm{if}\;c1>2\cdot mixPeriod\;\mathrm{and}\;C_{i}>2\cdot mixPeriod\\ 0, & \mathrm{otherwise} \end{array}\right.$$

The number of dimensions to be mixed (ms) is randomly drawn from the maximum boundary array according to the formation level via (41)

(41) $$ms=\left\{\begin{array}{ll} randi(mixSize[2]), & \mathrm{if}\;formation\_level=2\\ randi(mixSize[1]), & \mathrm{if}\;formation\_level=1 \end{array}\right.$$

During the mixing process, these selected dimensions of the source are swapped (crossover) with a 50% probability with either a random neighbor $x_{k}$ or the primal catalyzer Pbest according to (42).

(42) $$x_{i,j}=\left\{\begin{array}{ll} x_{k,j}, & \mathrm{if}\;rand>0.5\\ Pbest_{j}, & \mathrm{otherwise} \end{array}\right.$$

#### 4.1.4. HFO-1 Phase 4 & 5: Maturation & Saturation

In this periodic verification phase, it is tested whether the solutions in the population have reached homogeneity (epsilon_same) within the tolerance limits defined in (34). If all solutions have homogenized with Pbest, and the current Gbest has not improved for one full period compared to the leader of the previous period, pGbest, the honey is considered mature, and the swarm relocates to an entirely new area in the search space (newSite = true). To rapidly mature the new area, the current mixture is saturated with the best catalyzer, Gbest. In cases of prolonged stagnation, the mixing bounds mixSize(1) and mixSize(3) are automatically updated with random values drawn from the randPool created in (33) to rescue the algorithm from local optima.

### 4.2. MO-HFO

The fundamental HFO-1 algorithm directs the search process based on the cost associated with a single objective function. In contrast, multi-objective optimization (MOO) problems require the simultaneous optimization of multiple conflicting objectives. The MO-HFO algorithm incorporates a dual evaluation mechanism into the process while retaining the 5-phase search feature of HFO-1. In MO-HFO, the term “HFO” is used instead of “HFO-1” to avoid complexity in the algorithm’s naming. Within this framework, the intra-hive orientations of the bees, specifically selection and acceptance, are influenced by a scalarized proxy cost known as Honey Cost (HoneyCost). Additionally, high-quality trade-off solutions identified during the search are collected in an external Global Pareto Archive (REP) [48].

The 5-phase framework of the MO-HFO algorithm is presented in Algorithm 2 MO-HFO skeleton.
**Algorithm 2. MO-HFO (Multi-Objective Framework)**1-**//PHASE 1: INITIALIZATION**2-*Sources* ← initialize_sources()/**/**Positions are generated using HFO-1 Equation (31)3-initialize_variables()/**/**Timers are initialized using HFO-1 Equations (33)–(35)4-Evaluate Multiple Objectives $F(x_{i})$ and Penalty for all *Sources*/**/**Equation (43)5-*REP* ← Initialize Repository(*Sources*)/**/**Pareto Dominance check via Equations (44) and (45)6-Calculate scalar *HoneyCost* for all *Sources*/**/**Equation (46)7-*Gbest_HC* ← *Pbest_HC* ← best scalar *HoneyCost* from *Sources*8-**repeat**9-**//PHASE 2: HONEY FORMATION**10-
**for each ***source* of workers11-

*vi* ← local_search(*source*)//HFO-1 Equations (36) and (37)12-

Evaluate F(vi) and Penalty(vi)13-

*REP* ← Update Repository(*vi*)/**/**New candidate is submitted to the archive14-

**if ***HoneyCost(vi)* < *HoneyCost(source)* **then** replace *source* with *vi*/**/**Equation (47)15-
*P* ← selection_probabilities_based_on_HoneyCost(*Sources*)/**/**Adaptation of HFO-1 Equations (38) and (39)16-
**for each***onlooker*17-

*source* ← select_by_roulette(*P*)18-

*vi* ← local_search(*source*)19-

Evaluate F(vi) and Penalty(vi)20-

*REP* ← Update Repository(*vi*)21-

**if ***HoneyCost(vi)* < *HoneyCost(source)* **then** replace *source* with *vi*22-**//PHASE 3: MIXING**23-
**if** mixing phase condition is met/**/**HFO-1 Equation (40)24-

*vi* ← mix honey-forms/**/**HFO-1 Equations (41) and (42)25-

Evaluate F(vi) and Penalty(vi)26-

*REP* ← Update Repository(*vi*)27-
ensure the *Pbest* form is not corrupted28-**//PHASE 4 & 5: MATURATION & SATURATION**29-
**if ***Gbest_HC* is not improved for *maturePeriod*/**/**Scalar stagnation check30-

*Sources* ← initialize_sources()//Site matured (newSite = true)31-

*REP* ← Update Repository(*Sources*)32-
**else if** saturation condition is met33-

*Sources*[*mx*] ← inject *Gbest*34-

*REP* ← Update Repository(*Sources*[*mx*])35-
**if ***Gbest_HC* is not improved compared to *pGbest_HC*36-

change *mixSize* randomly37-**until** maximum iteration39-**return ***REP*//Final output is the Pareto Front

The new mathematical framework differentiating MO-HFO from the base algorithm and enabling multi-objective evolution is as follows:

#### 4.2.1. MO-HFO Phase 1: Initialization

After the candidate solutions are initialized in the search space, instead of a single cost, a Multi-Objective Cost Vector $F(x_{i})$ comprising M objective functions is calculated for the Decision Variable Vector $x_{i}$:

(43) $$F(x_{i})=[f_{1}(x_{i}),f_{2}(x_{i}),\ldots ,f_{M}(x_{i})]$$

##### Global Archive (Repository()) and Pareto Dominance

Before solutions are added to the external archive, they are subjected to the Pareto dominance rule [48,49]. The condition where solution $x_{1}$ dominates (is superior to) solution $x_{2}$ is denoted as $x_{1}≺x_{2}$, which requires two strict conditions to be satisfied simultaneously. For all objectives (m∈{1,2,…,M}), $x_{1}$ must be less than or equal to $x_{2}$ in (44), and it must be strictly less in at least one objective function in (45).

(44) $$\forall m\in \{1,2,\ldots ,M\},\;f_{m}(x_{1})\leq f_{m}(x_{2})$$

(45) $$\exists m\in \{1,2,\ldots ,M\},\;f_{m}(x_{1})<f_{m}(x_{2})$$

Solutions from the initial population that are not dominated by any other solution are appended to the REP archive.

##### Scalar Proxy Cost (HoneyCost())

To preserve the intra-hive orientations of the bees, the multi-dimensional vector $F(x_{i})$ is transformed into a scalar value by incorporating the constraint violation penalty value, $Penalty(x_{i})$, of the respective solution [25].

(46) $$HoneyCost(x_{i})=\sum_{m=1}^{M}f_{m}(x_{i})+2\cdot Penalty(x_{i})$$

The population leader (Pbest_HC) and the global leader (Gbest_HC) at initialization are determined as the solution possessing the minimum proxy cost.

#### 4.2.2. MO-HFO Phase 2 & 3: Honey Formation & Mixing

In these phases, each newly generated or mixed candidate solution ($v_{i}$) is subjected to two independent processes.

##### Archive Update

The $F(v_{i})$ values of the new solution are directly transmitted to the REP archive. If no solution in the archive dominates $v_{i}$ according to (44) and (45), this solution is included in the archive regardless of its scalar cost, thereby expanding the Pareto front.

##### Population Acceptance

For the candidate solution to replace the current source ($x_{i}$) in the main population, it is strictly conditioned upon an improvement in the scalar cost.

(47) $$HoneyCost(v_{i})<HoneyCost(x_{i})$$

Additionally, the roulette wheel selection probabilities of the onlooker bees are distributed based on these calculated HoneyCost fitness values rather than the standard objective function.

#### 4.2.3. MO-HFO Phase 4 & 5: Maturation & Saturation

The homogeneity checks via the floating-point equality utilized in HFO-1 are abandoned; the maturation process is entirely coupled to the observation of stagnation in the Gbest_HC value. If the scalar global leader of the system fails to improve over the maturePeriod, the corresponding region of the search space is considered mature, and the bees transition to a completely new site. Upon termination of the algorithm, instead of a single scalar solution, the REP archive comprising mutually non-dominated solutions is presented.

### 4.3. MO-EHFO

The scalarized proxy cost (HoneyCost) approach in the MO-HFO algorithm, while effective in guiding the search direction, remains limited in reflecting the “trade-off diversity” that is the fundamental nature of true Pareto optimality. The proposed MO-EHFO algorithm completely removes scalar costs from the decision mechanisms, establishing them strictly on the foundations of pure Pareto dominance, crowding distance [50], and logistic map-based chaos theory [51].

While the fundamental timing parameters and boundary control mechanisms of the algorithm are kept identical to HFO-1, the newly integrated enhanced functions and the updated 5-phase framework are presented in Algorithm 3 MO-EHFO skeleton.
**Algorithm 3. MO-EHFO (Enhanced Multi-Objective Framework)**1-**//PHASE 1: INITIALIZATION**2-*Sources* ← initialize_chaotic_sources()//Chaotic initialization via Equations (48) and (49)3-initialize_variables()//HFO-1 Equations (33)–(35)4-Evaluate Multiple Objectives $F(x_{i})$ and Penalty5-*REP* ← Initialize Repository_Smart(*Sources*)//Feasibility-first archive pruning6-*PBest_Pool* ← initialize_personal_archives(*Sources*)//Personal archives for each bee7-**repeat**8-update adaptive weight w(t)//Equations (51) and (52)9-**//PHASE 2: HONEY FORMATION**10-
**for each ***source* of workers11-

*Target* ← ArchiveLeader_CD(*REP*)/**/**Binary tournament leader selection via Equation (50)12-

*vi* ← LocalSearch_Enhanced(*source*, *Target*)/**/**Vectorial search via Equation (53)13-

Evaluate F(vi) and Penalty(vi)14-

**if ***HoneyCost(vi)* < *HoneyCost(source)* **then** replace *source* with *vi*/**/**Equation (47)15-

*REP*, *PBest_Pool* ← Update Repository_Smart(*vi*)16-
*P* ← ParetoSelectionProb(*Sources*)//Rank + CD based roulette via Equations (54) and (55)17-
**for each ***onlooker*18-

*source* ← select_by_roulette(*P*)19-

*Target* ← ArchiveLeader_CD(*REP*)20-

*vi* ← LocalSearch_Enhanced(*source*, *Target*)21-

Evaluate F(vi) and Penalty(vi)22-

*REP*, *PBest_Pool* ← Update Repository_Smart(*vi*)23-

**if** Pareto_Accept(*vi*, *source*) **then** replace *source* with *vi*24-**//PHASE 3: MIXING**25-
**if** mixing phase condition is met/**/**HFO-1 Equation (40)26-

**for each ***source*27-

*x_leader* ← PBestLeader(*PBest_Pool*)/**/**Personal elite leader via Equation (56)28-

*vi* ← mix honey-forms with *x_leader* and randoms/**/**HFO-1 crossover29-

Evaluate F(vi) and Penalty(vi)30-

*REP*, *PBest_Pool* ← Update Repository_Smart(*vi*)31-

**if not** Strict_Dominates(*source*, *vi*) **then** replace *source* with *vi*//Weak Pareto acceptance32-**//PHASE 4 & 5: MATURATION & SATURATION**33-
**if** no new non-dominated solution is admitted to REP **then** increment stagnation counter $c_{3}$//Archive growth-based stagnation tracking34-
**if **$c_{3}>maturePeriod$35-

change *mixSize* randomly//Automatic parameter tuning36-

**until** maximum evaluation limit is reached37-**until** maximum iteration38-**return ***REP*//Final output is the Pareto Front

The mathematical functions that make up MO-EHFO and are incorporated into the five phases are detailed below:

#### 4.3.1. MO-EHFO Phase 1: Initialization

##### Chaotic Initialization (Initialize_Chaotic_Sources())

Instead of uniform randomness, a logistic map-based chaotic ($z_{t}$) is utilized to achieve a more homogeneous distribution of the initial population within the search space [51]. The chaotic variable $z_{t}\in (0,1)$ at step t is updated via (48).

(48) $$z_{t+1}=4\cdot z_{t}\cdot (1-z_{t})$$

To avoid fixed points, the initial seed is selected as $z_{0}\in (0,1)\backslash \{0,0.25,0.5,0.75,1\}$. The position of the $i^{th}$ solution in the $j^{th}$ dimension is initialized via (49).

(49) $$x_{i,j}=x_{j}^{min}+z_{i}\cdot (x_{j}^{max}-x_{j}^{min})$$

##### Smart Archive Pruning (Repository_Smart())

When the global archive (REP) reaches its maximum capacity ($N_{A}$), the crowding distance (CD) of the solutions is calculated. Instead of standard random selection, the archive is pruned by retaining the $N_{A}$ solutions with the highest CD values, i.e., those in the sparsest regions.

##### Personal Pareto Archive (PBest_Pool)

For each bee (i), a personal archive ($PBest_{i}$) with a maximum capacity of 5 is initialized to store the non-dominated solutions discovered within its own search history. The additional memory required by these personal archives is bounded and small: with a per-individual capacity of five and a population of fifty, at most 250 solutions are stored at any time, of the same order as the single external archive, while each personal archive update operates on a five-element set and is therefore negligible relative to one objective function evaluation of the MO-OPF problem, which requires a full power flow solution. The diversity that these archives contribute to the recombination step is thus obtained at a negligible additional computational cost.

#### 4.3.2. MO-EHFO Phase 2: Honey Formation

##### Archive-Guided Leader Selection (ArchiveLeader_CD())

The target solution ($x^{target}$) that will guide the local search is determined not by a scalar global best [49,52], but through a binary tournament between two randomly selected candidate solutions ($a_{1},a_{2}$) from the REP, according to (50).

(50) $$x^{target}=arg\underset{s\in \{a_{1},a_{2}\}}{max}CD_{s}$$

where $CD_{s}$ is the crowding distance of the selected candidate.

##### Enhanced Local Search with Adaptive Weighting (LocalSearch_Enhanced())

Worker and onlooker bees employ a new search equation incorporating chaotic attraction ($z_{t}$) towards the target leader and a deterministically decreasing inertia weight (w(t)). This weight is not tuned from any fitness feedback, instead it follows a fixed, monotonically decreasing schedule that depends only on the ratio between the current number of function evaluations (FES) and the maximum evaluation limit (Max_FES), and is updated via (51).

(51) $$w(t)=w_{max}-\left(\frac{FES}{Max\_FES}\right)\cdot (w_{max}-w_{min})$$

Setting $w_{max}=1.0$ and $w_{min}=0.4$ ensures that the search is exploration-heavy initially and exploitation-heavy towards the end. The randomization factor $\phi_{j}$ is scaled via (52), where $r_{j}∼U(0,1)$, and the candidate solution $v_{i,j}$ is generated via (53).

(52) $$\phi_{j}=(2r_{j}-1)\cdot w(t)$$

(53) $$v_{i,j}=x_{i,j}+z_{t}\cdot (x_{j}^{target}-x_{i,j})+\phi_{j}\cdot (x_{i,j}-x_{k,j})$$

##### Feasibility-First Pareto Acceptance (Pareto_Accept())

The replacement of the current source ($x_{i}$) by the candidate solution ($v_{i}$) is tied to a feasibility-first Pareto rule [21,53]. Feasible solutions with no constraint violations are always preferred over infeasible ones. Although feasibility is prioritized during the acceptance step, this approach does not confine the search to conservative feasible regions. Instead, the handling of constraints is carried out through a gradual penalization process, as summarized in (57)–(60), rather than by directly rejecting the solution. This means that a candidate that is slightly infeasible but promising can still provide valuable direction information and can be addressed from the infeasible side. When integrated with leaders from the crowding-distance archive, which maintains boundary trade-offs, and an exploration-oriented early inertia weight, this strategy ensures that initially infeasible trade-offs are preserved rather than discarded prematurely. Consequently, the algorithm avoids converging on safe yet suboptimal feasible regions.

##### Pareto-Rank and Distance Selection (ParetoSelectionProb())

The roulette wheel probabilities ($P_{i}$) for source selection by onlooker bees are calculated by combining the Pareto Rank ($r_{i}$) obtained via non-dominated sorting and the $CD_{i}$ scores, according to (54) and (55).

(54) $$S_{i}=\frac{R_{max}-r_{i}+1}{R_{max}}+\frac{CD_{i}}{max(CD)}$$

(55) $$P_{i}=\frac{S_{i}}{\sum_{j=1}^{N_{pop}}S_{j}}$$

Here, $R_{max}$ represents the maximum rank value in the population, and $S_{i}$ denotes the composite selection score.

#### 4.3.3. MO-EHFO Phase 3: Mixing

##### Personal Pareto Leader (PBestLeader())

The primary catalyzer ($x_{pbest}^{leader}$) in the crossover process is determined by selecting the solution with the highest crowding distance [54] from the bee’s own personal archive ($PBest_{i}$), via (56).

(56) $$x_{pbest}^{leader}=arg\underset{s\in PBest_{i}}{max}CD_{s}$$

##### Weak Pareto Acceptance (Strict_Dominates())

The new solution formed as a result of the mixture is admitted to the population under a weak rule [55]. The hybrid is rejected only if the old solution “strictly” dominates the new one ($x_{i}≺v_{i}$); in all other cases, it is accepted, thereby injecting fresh genetic diversity into the population.

#### 4.3.4. MO-EHFO Phase 4 & 5: Maturation & Saturation

The removal of scalar costs has led to the assessment of the system’s maturity and saturation through Archive Growth Monitoring, which is based on admission events. If no new non-dominated solution is added to the REP archive within a specific iteration period, meaning that no newly generated solution qualifies as non-dominated and is thereby incorporated, the corresponding segment of the search space is considered mature. This prompts an increase in the stagnation counter, $c_{3}$. Once this counter exceeds the maturePeriod limit, the mixSize parameters are randomly resampled from the randPool defined in HFO-1 to help navigate away from local traps.

## 5. Results, Analysis and Comparative Study

In this section, the proposed MO-EHFO algorithm is tested and validated on the IEEE 30-bus test system integrated with RESs across nine different case studies. For each case study, the PDF parameters of the RESs, along with the technical and specific data of the power system, were applied as detailed in Table 1 and Table 2. Preliminary evaluations revealed that the standard MO-HFO algorithm falls short of reaching the true Pareto front and yields inadequate compromise solutions when compared to the robust and well-established NSGA-II algorithm. The MO-EHFO algorithm was specifically developed to address and overcome these limitations. By taking into account the varying objective functions across the case studies, the performance of the proposed MO-EHFO is presented through visual and numerical data and subjected to an in-depth analysis. Furthermore, to establish a fair and transparent benchmark, the optimal solution values of the objective functions were compared against selected state-of-the-art algorithms from the literature. In presenting the Pareto fronts, meticulous attention was given to reflecting the best solution points of comparable algorithms evaluated under identical conditions. Table 3 provides an overview of the case studies related to MO-OPF issues in this paper.

### 5.1. MO-OPF Studies

To evaluate the proposed algorithm’s performance, nine case studies (five bi-objective, three tri-objective, and one quad-objective) were conducted on the modified IEEE 30-bus system, as detailed below.

#### 5.1.1. Bi-Objective MO-OPF Studies

The success of the proposed MO-EHFO algorithm for the MO-OPF problem is verified and compared against our previously developed MO-HFO algorithm and the robust NSGA-II. The global best compromise solutions of the selected algorithms for the five case studies are compared with those of MO-EHFO in Table 4 and Table 5.

**Figure 2:**
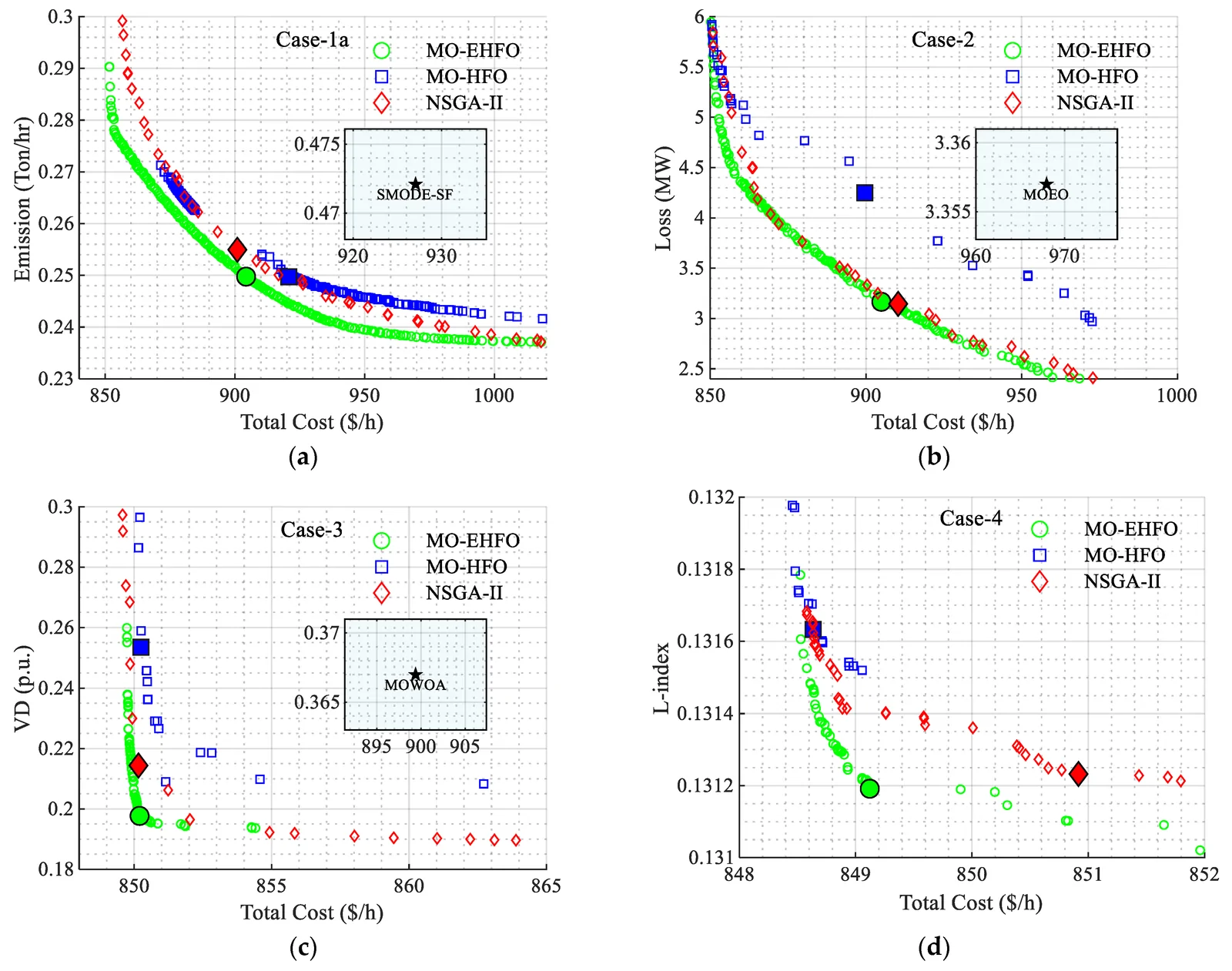
Pareto-optimal fronts obtained using the MO-EHFO, MO-HFO, and NSGA II algorithms: (**a**) Case 1a, (**b**) Case 2, (**c**) Case 3, and (**d**) Case 4. The larger markers denote the best compromise solution obtained by each algorithm.

As shown in Figure 2a–d, MO-EHFO converges much closer to the true Pareto front compared to the NSGA-II and MO-HFO algorithms. Figure 2a demonstrates the superior performance of MO-EHFO, which yields a remarkably smooth, dense, and gap-free continuous front. By achieving the best compromise solution of $904.3035/h and 0.2498 ton/h, MO-EHFO outperforms both NSGA-II and MO-HFO.

The compromise solution for cost and emission values reported in reference [25] ($927.049/h and 0.4721 ton/h) falls outside the scaled limits of Figure 2a. Therefore, this solution was brought inward and is presented in a separate inset within the graph. In this plot, MO-HFO exhibits a successful performance comparable to NSGA-II. However, while MO-HFO produced clustered solution sets, the proposed algorithm distributed these solutions toward the edges, creating a more uniform spread.

Figure 2b indicates that the proposed MO-EHFO is significantly ahead of the existing literature. The best compromise value obtained by the Multi-Objective Equilibrium Optimizer (MOEO) [31] ($967.977/h and 3.357 MW) falls far outside the scaled display; thus, it was also moved inward and shown in a separate inset. Compared to NSGA-II’s strong solution of 3.1453 MW for power loss, MO-EHFO achieved 3.1658 MW, demonstrating a solution that sacrifices a marginal amount of power loss to provide a substantially higher rate of improvement on the cost side. In Case 2, the MO-EHFO algorithm converges to the Pareto front faster and more stably, providing a more homogeneous solution distribution along the entire front. While NSGA-II lags behind MO-EHFO, it was observed that the MO-HFO algorithm exhibited limited performance in both convergence and diversity.

The irregular Pareto distributions seen in Figure 2c,d are associated with the non-convex nature and the complex mathematical characteristics of the selected objective functions, such as voltage deviation and L-index. Due to the inherent nature of the problem, the geometries of the Pareto fronts lacked uniformity in terms of solution set diversity. In Figure 2c,d, MO-EHFO achieved faster convergence in the low-cost region. Although MO-HFO offers a broader search space, it exhibited a discontinuous distribution on the Pareto front. This can be attributed to the strong exploration capability of the MO-HFO algorithm, coupled with the inability of the solutions to drop below certain thresholds for the selected objective functions. NSGA-II, despite its diversity mechanisms, failed to achieve a homogeneous spread along the front due to the non-convex structure of the objective functions.

In Case 3, regarding the optimization of cost and voltage deviation functions, MO-EHFO produced an excellent solution set with a best compromise point of $849.9376/h and 0.2078 p.u. In Figure 2c, the best compromise value of $899.393/h and 0.36749 p.u. obtained by the Multi-Objective Whale Optimization Algorithm (MOWOA) [31] falls far outside the scaled screen, thus it had to be presented as an inset. Finally, in Figure 2d, which illustrates Case 4, it is observed that the Pareto solutions intensively attempt to minimize the cost; however, while improving the L-index, the solutions become sparse due to the mathematical characteristics of the function and the saturation effect of reaching maximum improvement.

#### 5.1.2. Triple-Objective MO-OPF Studies

**Figure 3:**
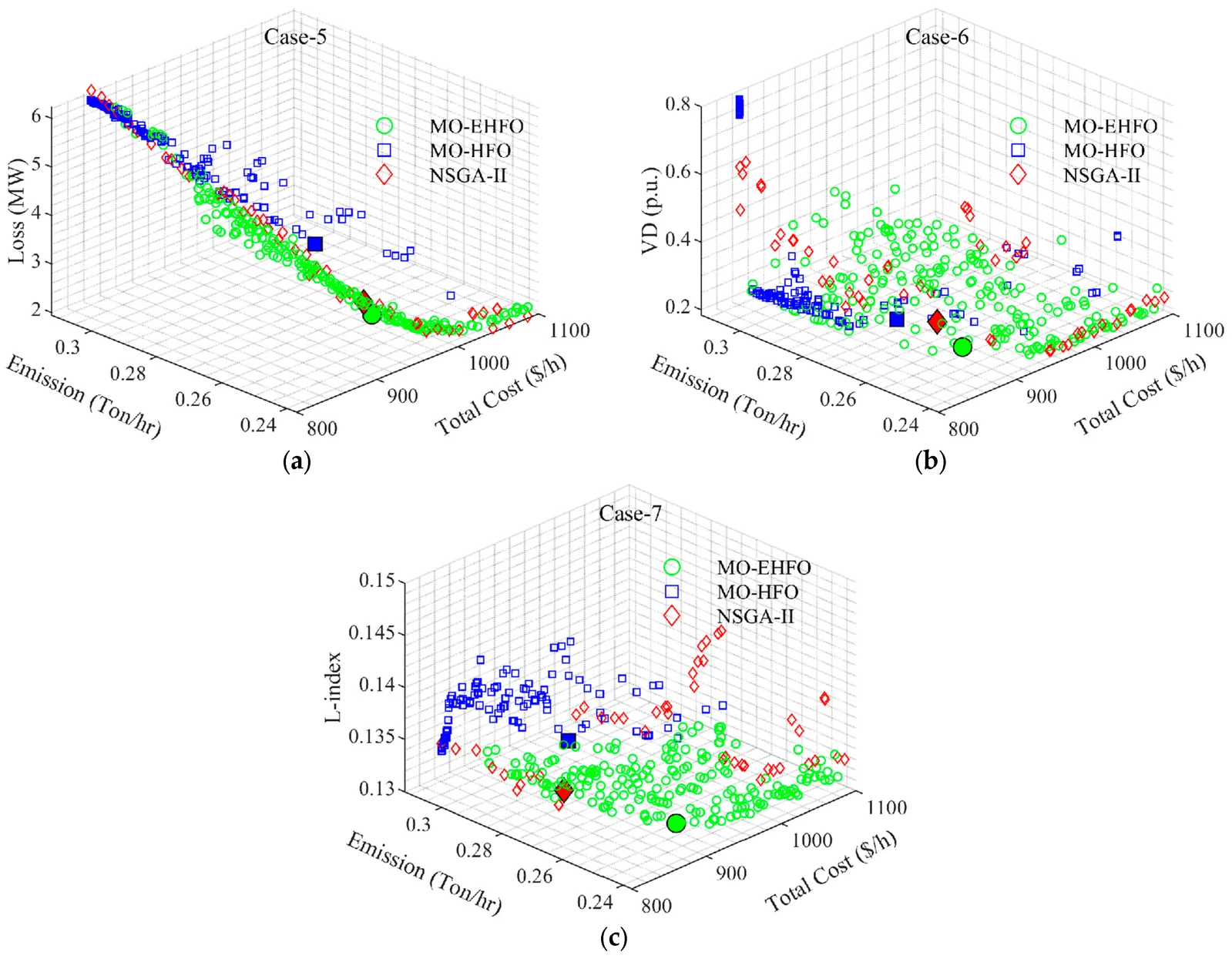
Pareto-optimal fronts obtained using the MO-EHFO, MO-HFO, and NSGA II algorithms: (**a**) Case 5, (**b**) Case 6, and (**c**) Case 7. The larger markers denote the best compromise solution obtained by each algorithm.

The global best compromise solutions of the selected algorithms for the tri-objective case studies are compared with the proposed MO-EHFO in Table 6. As depicted in Figure 3a, MO-EHFO exhibits a highly balanced Pareto distribution and strong convergence characteristics. In contrast, the effects of NSGA-II’s non-dominated sorting and crowding distance-based selection mechanisms are visually apparent; while it concentrates heavily in certain regions, it provides sparse and irregular solutions at the extremes of the Pareto front. For MO-HFO, this irregularity causes instability and creates a scattered appearance. MO-EHFO, on the other hand, establishes an optimal exploration-exploitation balance. Driven by its robust search capability and strong convergence characteristics, it yields both a smoothly distributed Pareto front and an outstanding compromise point.

Based on these final compromise values Table 8, MO-EHFO not only demonstrates superior performance with a more balanced and high-quality solution point compared to NSGA-II and MO-HFO, but it also captures a significantly better solution than the best compromise value reported by the Multi-Objective Ions Motion Optimization (MOIMO) [31] algorithm ($973.465/h, 0.141 ton/h, and 3.282 MW) for the same objective functions in the literature.

As observed in Figure 3b, the proposed MO-EHFO algorithm covers a remarkably broader section of the Pareto front in this complex tri-objective optimization problem, offering exceptionally high solution diversity. The standard MO-HFO algorithm converges prematurely and clusters densely in a strictly limited region, thereby drastically reducing Pareto diversity. Meanwhile, NSGA-II generates disjointed and disconnected solutions, exhibiting particular instability in optimizing the non-convex voltage deviation objective. The proposed MO-EHFO, with its superior exploration capability, searches the entire solution space with extraordinary coverage, achieving both a perfect diversity spread across the Pareto front and capturing the optimal compromise point in a position significantly better than its competitors and those reported in [31].

Evidently from Figure 2 and Figure 3, MO-EHFO, equipped with chaotic mutation and pure Pareto dominance, crowding distance-based smart archive pruning, adaptive weighting, and feasibility-first acceptance strategies, achieves superior compromise points compared to both the selected baseline algorithms and the state-of-the-art algorithms reported in the literature under identical conditions.

#### 5.1.3. Quad-Objective MO-OPF Studies

In the quad-objective case study 8, the best compromise solutions of NSGA-II, MO-HFO, and MO-EHFO are presented in Table 7. Among these, the best compromise solution of the MO-EHFO algorithm exhibited superior performance across all objectives compared to NSGA-II, MO-HFO, and other algorithms reported in the literature, namely MOIMO [31], MOEO [31], Multi-Objective Grey Wolf Optimizer (MOGWO) [31], and MOWOA [31]. Similar to previous case studies, MO-HFO achieved a lower cost objective, whereas NSGA-II resulted in the highest cost value at $965.7267/h. When comparing the voltage deviation values among all algorithms, MO-EHFO demonstrated excellent performance by attaining the lowest value of 0.2323 p.u. for this specific objective.

### 5.2. Detailed Evaluation of Results in Terms of Security and Operational Constraints

In addition to ensuring the operational continuity and stability of power systems, it is imperative that all system constraints are strictly respected to guarantee technical and secure operation. With the integration of RESs, the multi-objective OPF problem has become a highly non-linear, discrete, and multi-modal optimization challenge. In this study, all operational security margins, technical limits, and system constraints were strictly maintained. Any candidate Pareto solutions that violated the specified boundaries were systematically discarded using an effective penalty mechanism, thereby ensuring the generation of exclusively feasible and secure solution sets.

In addition to maintaining generator voltages and active power outputs within their desired secure limits, it is essential to consider POZs. The applied penalty term must prevent the operating point of the generator from entering the lower or upper bands of the POZs. In this context, a penalty term must also be applied to control the real power generation output at the slack bus ($PG_{slack}$). While keeping the system within secure operating limits, the load bus voltages ($V_{L}$), generator reactive power outputs ($Q_{G}$), and transmission line loadings ($S_{TL}$) act as critical security constraint criteria. As demonstrated in the subsequent equations, secure solution sets that avoid undesirable operating points were obtained by incorporating a penalty value into the objective function.

The objective function value, f(x), is updated through sequential operations via (57)–(60) to penalize any boundary violations. If the slack variable violates its defined limits, it is heavily penalized with a weight factor (ξ) equal to 1000, as formulated in (58).

(57) $$\left[P_{G_{slack}},Y\right]=PowerFlow\left(u\right)$$

(58) $$P_{G_{slack}}=\xi .P_{G_{slack}}\cdot \left(P_{G_{slack}}>Ub_{G_{slack}}\vee P_{G_{slack}}<Lb_{G_{slack}}\right)$$

(59) $$Penalty\left(Y\right)=w\cdot \sum_{i=1}^{n}\left[\left|Ub_{i}-Y_{i}\right|\cdot \left(Y_{i}>Ub_{i}\right)+\left|Lb_{i}-Y_{i}\right|\cdot \left(Y_{i}<Lb_{i}\right)\right]$$

Here, the PowerFlow(x) function is defined according to [56], where $Ub_{G_{slack}}$ and $Lb_{G_{slack}}$ represent the upper and lower bounds of the slack variable, respectively, and Y denotes the vector of security constraint parameters. Furthermore, $Ub_{i}$ and $Lb_{i}$ correspond to the upper and lower bounds of the i-th constraint parameter, and w represents the penalty coefficient, which is determined empirically for each multi-objective function definition. The objective function value, f(x), utilized within the algorithm is then updated using (60), incorporating the slack variable adjustments defined in (57) and applying an additional penalty for any bound violations.

(60) $$f\left(x\right)=f\left(x\right)+Penalty\left(Y\right)$$

**Figure 4:**
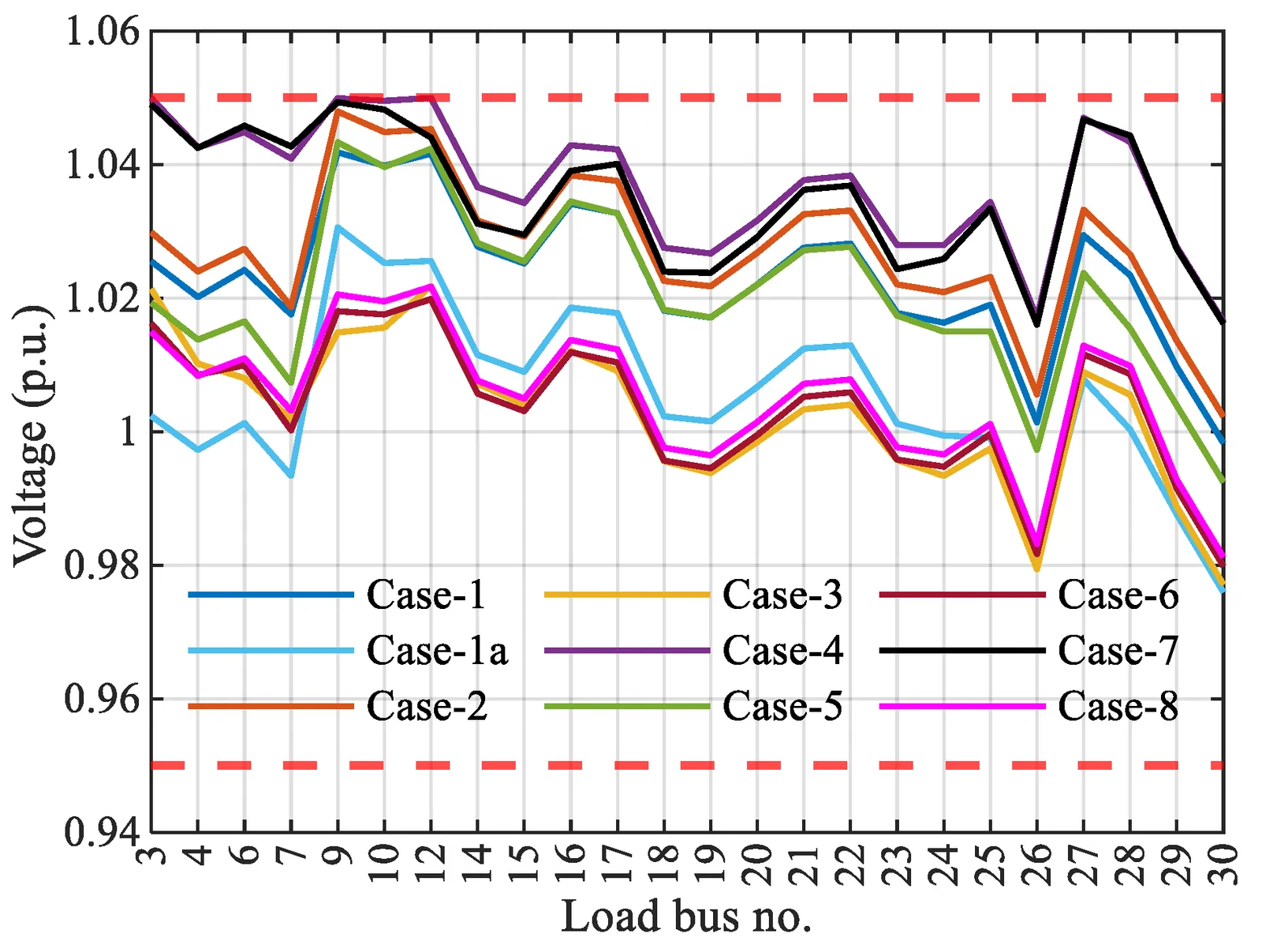
Bus voltage profiles for all cases using MO-EHFO.

In this section, we transparently present the data regarding these two constraints, which are frequently overlooked or inadequately reported in many existing studies in the literature. Figure 4 illustrates that the load bus voltages are successfully maintained within the permissible range of [0.95–1.05] p.u. It can be clearly observed that the voltages in Case 4 and Case 7 approach the upper boundary. This outcome is both normal and expected in case studies where the primary focus is on enhancing voltage stability. Furthermore, the results confirming our strict adherence to the reactive power constraints of the generators, with absolutely no violations of the lower and upper limits, are detailed at the bottom of Table 4, Table 5, Table 6 and Table 7.

An examination of the table results reveals that generator reactive power outputs shift to negative values at certain buses. This characteristic has positively contributed to the minimization of reactive power losses, and it is observed that the MO-EHFO algorithm is capable of achieving lower $Q_{loss}$ values by operating specific generators at negative or low reactive power levels. For instance, in Case 1, the $Q_{loss}$ value for MO-EHFO was attained at a significantly lower level of −0.2544 MVAr compared to other methods.

### 5.3. Detailed Comparative Analysis

Table 8 presents the best compromise solutions obtained for different OPF scenarios and provides comparative knowledge between the proposed approach and several well-known algorithms reported in the literature, which were selected to ensure a fair comparison under identical operating conditions. A careful evaluation of the tabulated results indicates that the best compromise solutions achieved for various multi-objective OPF scenarios highlight distinct behavioral characteristics of the considered algorithms. Specifically, the algorithms referenced from the literature yield significantly higher generation cost values across most cases, demonstrating limited effectiveness in minimizing the overall operating cost of the system. However, these algorithms occasionally achieve superior results for specific individual objectives in certain scenarios; for example, relatively lower emission values are observed in Case 5.

**Table 8:** Comparison of the proposed algorithm with the literature.

Case No.	Algorithms	Literature Variables Limits	$F_{CT}$	$F_{E}$	$F_{PL}$	$F_{VD}$	$F_{VSI}$
		$\left[V_{k,min}^{G}-V_{k,max}^{G}\right]$, $\left[V_{k,min}^{L}-V_{k,max}^{L}\right],N$					
Case-1	MOEO [31]	$\left[0.95-1.1\right]$$,\;\;\left[0.95-1.05\right]$ , 11	923.821	0.574	-	-	-
	MOWOA [31]	$\left[0.95\;-\;1.1\right]$, $\left[0.95\;-\;1.05\right]$, 11	932.679	0.424	-	-	-
	MOGWO [31]	$\left[0.95\;-\;1.1\right]$, $\left[0.95\;-\;1.05\right]$, 11	930.661	0.432	-	-	-
	MOIMO [31]	$\left[0.95\;-\;1.1\right]$, $\left[0.95\;-\;1.05\right]$, 11	925.810	0.534	-	-	-
	NSGA-II	$\left[0.95\;-\;1.1\right]$, $\left[0.95\;-\;1.05\right]$, 11	890.3921	0.2561	-	-	-
	MO-HFO	$\left[0.95\;-\;1.1\right]$, $\left[0.95\;-\;1.05\right]$, 11	893.2257	0.2603	-	-	-
	**MO-EHFO**	$\left[0.95\;-\;1.1\right]$, $\left[0.95\;-\;1.05\right]$, 11	**901.7422**	**0.2506**	-	-	-
Case-1a	MOEA/D-SF [25]	$\left[0.95\;-\;1.1\right]$, $\left[0.95\;-\;1.05\right]$, 11	919.040	0.6221	-	-	-
	SMODE-SF [25]	$\left[0.95\;-\;1.1\right]$, $\left[0.95\;-\;1.05\right]$, 11	927.049	0.4721	-	-	-
	NSGA-II	$\left[0.95\;-\;1.1\right]$, $\left[0.95\;-\;1.05\right]$, 11	900.9256	0.2549	-	-	-
	MO-HFO	$\left[0.95\;-\;1.1\right]$, $\left[0.95\;-\;1.05\right]$, 11	920.7629	0.2497	-	-	-
	**MO-EHFO**	$\left[0.95\;-\;1.1\right]$, $\left[0.95\;-\;1.05\right]$, 11	**904.3035**	**0.2498**	-	-	-
Case-2	MOEO [31]	$\left[0.95\;-\;1.1\right]$, $\left[0.95\;-\;1.05\right]$, 11	967.977	-	3.357	-	-
	MOWOA [31]	$\left[0.95\;-\;1.1\right]$, $\left[0.95\;-\;1.05\right]$, 11	967.066	-	3.418	-	-
	MOGWO [31]	$\left[0.95\;-\;1.1\right]$, $\left[0.95\;-\;1.05\right]$, 11	975.684	-	3.104	-	-
	MOIMO [31]	$\left[0.95\;-\;1.1\right]$, $\left[0.95\;-\;1.05\right]$, 11	967.879	-	3.363	-	-
	NSGA-II	$\left[0.95\;-\;1.1\right]$, $\left[0.95\;-\;1.05\right]$, 11	910.3383	-	3.1453	-	-
	MO-HFO	$\left[0.95\;-\;1.1\right]$, $\left[0.95\;-\;1.05\right]$, 11	899.6483	-	4.2484	-	-
	**MO-EHFO**	$\left[0.95\;-\;1.1\right]$, $\left[0.95\;-\;1.05\right]$, 11	**904.9426**	-	**3.1658**	-	-
Case-3	MOEO [31]	$\left[0.95\;-\;1.1\right]$, $\left[0.95\;-\;1.05\right]$, 11	901.303	-	-	0.349	-
	MOWOA [31]	$\left[0.95\;-\;1.1\right]$, $\left[0.95\;-\;1.05\right]$, 11	899.393	-	-	0.367	-
	MOGWO [31]	$\left[0.95\;-\;1.1\right]$, $\left[0.95\;-\;1.05\right]$, 11	902.587	-	-	0.345	-
	MOIMO [31]	$\left[0.95\;-\;1.1\right]$, $\left[0.95\;-\;1.05\right]$, 11	899.613	-	-	0.358	-
	NSGA-II	$\left[0.95\;-\;1.1\right]$, $\left[0.95\;-\;1.05\right]$, 11	850.1572	-	-	0.2144	-
	MO-HFO	$\left[0.95\;-\;1.1\right]$, $\left[0.95\;-\;1.05\right]$, 11	850.2454	-	-	0.2535	-
	**MO-EHFO**	$\left[0.95\;-\;1.1\right]$, $\left[0.95\;-\;1.05\right]$, 11	**850.1972**	-	-	**0.1977**	-
Case-4	NSGA-II	$\left[0.95\;-\;1.1\right]$, $\left[0.95\;-\;1.05\right]$, 11	850.9161	-	-	-	0.1312
	MO-HFO	$\left[0.95\;-\;1.1\right]$, $\left[0.95\;-\;1.05\right]$, 11	848.6354	-	-	-	0.1316
	**MO-EHFO**	$\left[0.95\;-\;1.1\right]$, $\left[0.95\;-\;1.05\right]$, 11	**849.1218**	-	-	-	**0.1312**
Case-5	MOEO [31]	$\left[0.95\;-\;1.1\right]$, $\left[0.95\;-\;1.05\right]$, 11	975.734	0.139	3.289	-	-
	MOWOA [31]	$\left[0.95\;-\;1.1\right]$, $\left[0.95\;-\;1.05\right]$, 11	964.521	0.213	3.560	-	-
	MOGWO [31]	$\left[0.95\;-\;1.1\right]$, $\left[0.95\;-\;1.05\right]$, 11	965.231	0.172	3.579	-	-
	MOIMO [31]	$\left[0.95\;-\;1.1\right]$, $\left[0.95\;-\;1.05\right]$, 11	973.465	0.141	3.282	-	-
	NSGA-II	$\left[0.95\;-\;1.1\right]$, $\left[0.95\;-\;1.05\right]$, 11	920.4789	0.2462	3.1610	-	-
	MO-HFO	$\left[0.95\;-\;1.1\right]$, $\left[0.95\;-\;1.05\right]$, 11	904.8058	0.2574	4.1682	-	-
	**MO-EHFO**	$\left[0.95\;-\;1.1\right]$, $\left[0.95\;-\;1.05\right]$, 11	**923.7084**	**0.2445**	**2.9262**	-	-
Case-6	MOEO [31]	$\left[0.95\;-\;1.1\right]$, $\left[0.95\;-\;1.05\right]$, 11	896.392	2.055	-	0.519	-
	MOWOA [31]	$\left[0.95\;-\;1.1\right]$, $\left[0.95\;-\;1.05\right]$, 11	897.039	2.671	-	0.484	-
	MOGWO [31]	$\left[0.95\;-\;1.1\right]$, $\left[0.95\;-\;1.05\right]$, 11	918.946	0.838	-	0.416	-
	MOIMO [31]	$\left[0.95\;-\;1.1\right]$, $\left[0.95\;-\;1.05\right]$, 11	926.204	1.052	-	0.492	-
	NSGA-II	$\left[0.95\;-\;1.1\right]$, $\left[0.95\;-\;1.05\right]$, 11	887.6461	0.2591	-	0.2831	-
	MO-HFO	$\left[0.95\;-\;1.1\right]$, $\left[0.95\;-\;1.05\right]$, 11	870.9783	0.2677	-	0.2723	-
	**MO-EHFO**	$\left[0.95\;-\;1.1\right]$, $\left[0.95\;-\;1.05\right]$, 11	**897.3254**	**0.2535**	-	**0.2196**	-
Case-7	NSGA-II	$\left[0.95\;-\;1.1\right]$, $\left[0.95\;-\;1.05\right]$, 11	859.6125	0.2743	-	-	0.1330
	MO-HFO	$\left[0.95\;-\;1.1\right]$, $\left[0.95\;-\;1.05\right]$, 11	874.0991	0.2764	-	-	0.1370
	**MO-EHFO**	$\left[0.95\;-\;1.1\right]$, $\left[0.95\;-\;1.05\right]$, 11	**909.2190**	**0.2494**	-	-	**0.1313**
Case-8	MOEO [31]	$\left[0.95\;-\;1.1\right]$, $\left[0.95\;-\;1.05\right]$, 11	954.019	0.256	4.543	0.716	-
	MOWOA [31]	$\left[0.95\;-\;1.1\right]$, $\left[0.95\;-\;1.05\right]$, 11	943.289	0.909	8.320	0.518	-
	MOGWO [31]	$\left[0.95\;-\;1.1\right]$, $\left[0.95\;-1.05\right]$, 11	966.322	0.311	5.604	0.595	-
	MOIMO [31]	$\left[0.95\;-\;1.1\right]$, $\left[0.95\;-\;1.05\right]$, 11	951.374	0.297	4.830	0.423	-
	NSGA-II	$\left[0.95\;-\;1.1\right]$, $\left[0.95\;-\;1.05\right]$, 11	965.7267	0.2427	3.1174	0.3132	-
	MO-HFO	$\left[0.95\;-\;1.1\right]$, $\left[0.95\;-\;1.05\right]$, 11	901.0483	0.2565	3.8930	0.2384	-
	**MO-EHFO**	$\left[0.95\;-\;1.1\right]$, $\left[0.95\;-\;1.05\right]$, 11	**920.7131**	**0.2459**	**3.2316**	**0.2323**	-

Bold values indicate the best result.

Although the MO-HFO algorithm achieves lower generation cost values in many instances, it generally produces higher values for other objectives when compared to NSGA-II and the proposed MO-EHFO algorithm. While this trend indicates that the MO-HFO algorithm tends to direct the search process toward economically favorable regions of the Pareto space, its performance regarding other operational metrics remains relatively weaker. On the other hand, the NSGA-II algorithm provides relatively balanced results across the objectives; however, its best compromise solutions cannot always simultaneously achieve the optimal minimum values for all objective functions. In contrast, the proposed MO-EHFO algorithm demonstrates a more effective compromise among the conflicting objectives by maintaining highly competitive cost levels while simultaneously improving emission, power loss, voltage deviation, and voltage stability performance in the vast majority of the studied cases.

For completeness, the control settings of the literature algorithms whose compromise solutions are quoted for comparison in Table 8 are those reported in their source studies. In [31], the MOEO, MOWOA, MOGWO and MOIMO results were obtained with a population size of 30 evolved over 500 generations, the remaining algorithm-specific parameters being set to the defaults recommended in each method’s original paper. In [25], the MOEA/D-SF and Summation-based Multi-Objective Differential Evolution with Superiority of Feasible solutions (SMODE-SF) results used a population of 200 with a maximum of 100,000 function evaluations, a neighborhood size T = 0.15 N, a neighbor-update probability of 0.9, a crossover rate CR = 0.9 and a mutation (scaling) factor F = 0.5, averaged over 21 independent runs.

### 5.4. Seasonal Scenario-Based Validation of the MO-EHFO Algorithm

This section discusses the validity of the proposed algorithm using scenarios in which renewable energy sources are configured to represent specific seasonal periods. Using the Weibull, lognormal, and Gumbel distributions, whose parameters are given in Section 2.2 and Table 1, 8000 Monte Carlo scenarios were generated, representing different combinations of wind speed, solar radiation, and water flow conditions throughout the year. This large set of 8000 scenarios was reduced to 25 representative scenarios using a scenario-reduction technique [57]. These 25 scenarios provide a good approximation of the original 8000 scenarios in terms of their probabilistic values. The Case 1 and Case 1a studies, which jointly consider the use of RESs in terms of emissions and costs on the power system, were revisited here and evaluated using the 25 scenarios. This makes it easier to understand the economic and environmental impacts that the electrical power generated by RESs at different times of the year will have on the grid. The data for the selected scenarios and the optimal trade-off points for each scenario are presented in Table 9. Scenarios 6 and 24 have the lowest probability values, at 0.0001. In these scenarios, the highest solar irradiance values caused the PV generators at busbars 8 (50 MW) and 11 (45 MW) to operate at full power. On the other hand, the wind turbine installed at bus 5 generated 53.8522 MW and 39.5343 MW in the two scenarios and, combined with the high water-flow rates, achieved the best compromise emission values in both scenarios. In Case 1, 0.2387 ton/h and 0.2415 ton/h for scenarios 6 and 24, respectively, and in Case 1a, 0.2385 ton/h and 0.2426 ton/h, respectively, significantly reducing the emission values for both cases.

In particular, the emission values in Scenario 6 are among the lowest in the table. The impact of high penetration of renewable energy sources on this result is evident. Although the increase in the production potential of the RESs units has led to positive environmental outcomes, it has not resulted in an optimistic economic picture. In Scenarios 6 and 24, the increase in RES production has also led to higher costs. Restrictions on the operating zones of thermal generators subject to POZ have caused a further increase in production costs. The cost values for the best compromise points in Case 1 were significantly higher for Scenarios 6 and 24 at 1247.5994 $/h and 1180.3775 $/h, respectively, and in Case 1a at 1249.5081 and 1179.0140 $/h. In particular, the cost values for these scenarios rank among the top five highest values in the table. Scenario No. 8, which has the highest probability, yielded a more optimistic cost result. Although the wind speed and flow rates for this scenario are high, the low level of solar irradiance reduced the contribution of solar power plants to power generation and increased emissions. Among the best compromise values obtained from the 25 scenarios, the best and worst were calculated using the Euclidean method and correspond to scenarios 18 and 17, respectively. Despite the POZ constraints, the proposed algorithm maintains the same ranking for both the easiest and most challenging operating conditions. If the search process were highly stochastic or unstable, different scenarios would be expected to produce the minimum and maximum compromise distances after the introduction of POZ constraints. This behavior demonstrates that MO-EHFO exhibits a high degree of robustness and consistency.

**Figure 5:**
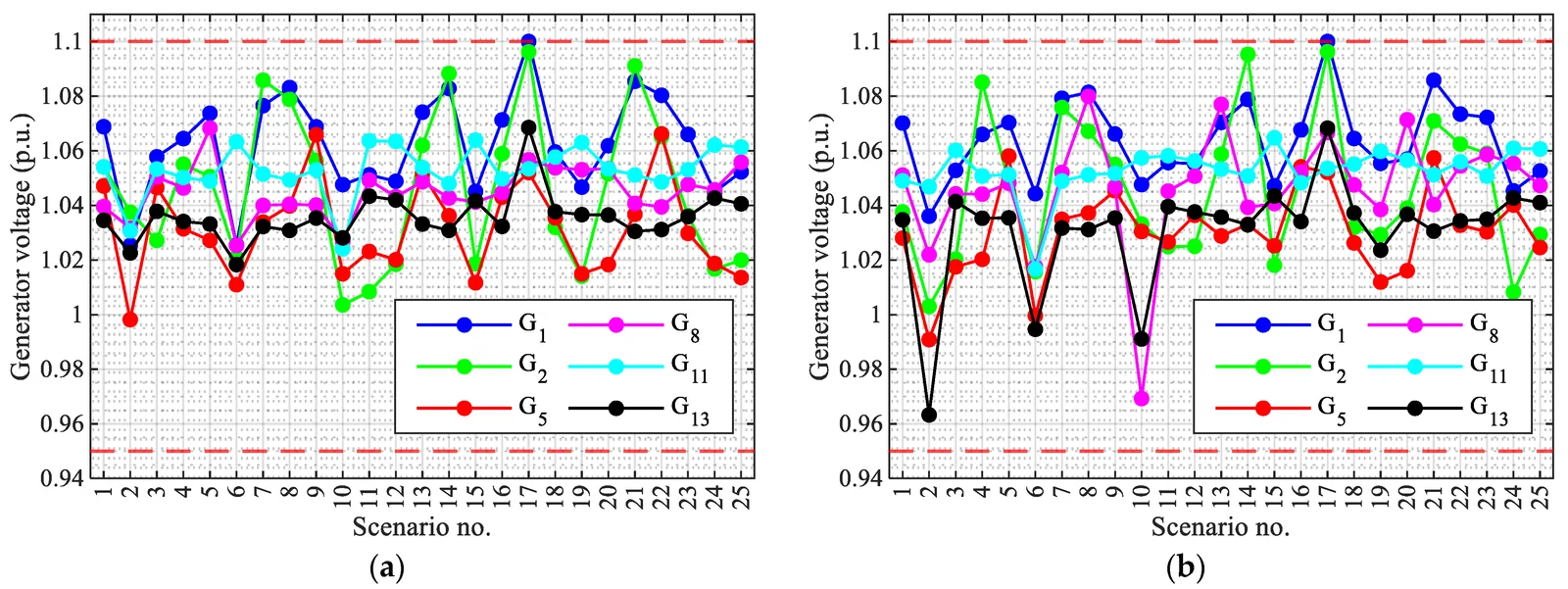
Generator voltages for reduced scenarios: (**a**) Case 1 and (**b**) Case 1a. The red dashed lines represent the lower and upper generator voltage limits (0.95–1.10 p.u.).

Figure 5 shows that in the Case 1 and Case 1a studies, the generator voltages for each scenario were successfully maintained within the 0.95–1.1 p.u. range. In Case 1, the generator voltages fluctuate slightly more freely, whereas in Case 1a (with POZ), the voltage profiles are more balanced and concentrated within a narrower band and at relatively lower levels. In Scenario 17, which has the worst compromise point, the voltage levels of thermal generators 1 and 2 reach their maximum values in both cases, highlighting the need for voltage support. The results demonstrate that the proposed MO-EHFO algorithm consistently produces feasible, well-distributed Pareto-optimal solutions across a wide range of seasonal renewable generation conditions. Therefore, the algorithm is not limited to a single-day operating point and maintains its optimization capability across different renewable generation patterns throughout the year.

### 5.5. Numerical Comparisons

To demonstrate the superiority of the proposed MO-EHFO algorithm on the IEEE 30-bus test system, a metric evaluation was performed using the basic MO-HFO algorithm, which evolved from a single-objective to a multi-objective function, and the NSGA-II algorithm, which is well established in the literature [40]. The same model, control variable limits, and constraint management strategy were used for each method on the test system. Thus, any performance differences reflect the search behavior of the algorithms themselves rather than the problem formulation. Four bi-objective scenarios were examined as cost–emission (Case-1), cost–loss (Case-2), cost–VD (Case-3), and cost–L-index (Case-4). Because population-based metaheuristic algorithms are stochastic, each pair of cases was run 20 times, with runs statistically independent of one another. The resulting Pareto archives were then evaluated using three complementary quality indicators, namely spread (Δ), hypervolume (HV), and inverted generational distance (IGD), which jointly capture the diversity, coverage, and convergence of an approximation set [58,59].

Since the analytical form of the true Pareto front is often unknown in complex power system problems such as the IEEE test systems, the IGD and spread indicators have been evaluated using a widely adopted approach in the literature. In this context, all solutions of the three compared algorithms were gathered into a single pool, non-dominant points in this pool were filtered to obtain a common artificial reference front, and the indicators were calculated relative to this front. To ensure consistency across comparisons, the Pareto sets obtained in each scenario were first mapped onto a common normalized objective space; then, the best compromise solution from each independent run was selected as the archive element with the smallest normalized Euclidean distance to the ideal point. The control parameters of the compared algorithms are summarized in Table 10; the values given for the proposed MO-EHFO and the basic MO-HFO are based on the algorithmic configuration adopted in this study, whereas the NSGA-II operators use the traditional simulated binary crossover (SBX) and polynomial mutation setups [59].

#### 5.5.1. Spread Indicator (Δ)

The spread metric is employed to quantitatively evaluate the homogeneity of non-dominant solutions along the approximation front and to assess how well the extreme regions are represented. For the set Ω obtained in an m-objective problem, this metric is defined according to Equations (61)–(63), considering the intervals between successive solutions and the distance of the boundary solutions $E_{i\;}$ from the front [58,60].

(61) $$∆=\frac{\sum_{i=1}^{m}d\left(E_{i},Ω\right)+\sum_{X\in Ω}\left|d\left(X,Ω\right)-d_{avg}\right|}{\sum_{i=1}^{m}d\left(E_{i},Ω\right)+(\left|Ω\right|-k)d_{avg}}$$

(62) $$d(X,Ω)=\underset{Y\in Ω,Y\neq X}{\min}\left\|F\left(X\right)-F(Y)\right\|$$

(63) $$d_{avg}=\frac{1}{\left|Ω\right|}\sum_{X\in Ω}d(X,Y)$$

where d(X,Ω) represents the Euclidean distance to the nearest neighbor of an X solution within the set Ω, while $d_{avg}$ is the arithmetic mean of these nearest neighbor distances, and $\left|Ω\right|$ indicates the number of elements in the set. A small Δ value indicates that the solutions are distributed evenly and without gaps, covering even the extreme regions, whereas a large value reflects clustering or gaps on the frontier. The first four columns of Table 11 present the spread values obtained from 20 independent trials for each scenario, including the average, standard deviation, minimum, and maximum. The best result in each row is highlighted in bold. When Case-4 is examined, the spread metric could not be computed for the MO-HFO algorithm because its non-dominated solution set contained fewer than two members. This is because the spread indicator, by definition, requires at least two solutions to be evaluated. This outcome clearly highlights how challenging the cost–L-index optimization problem is.

**Figure 6:**
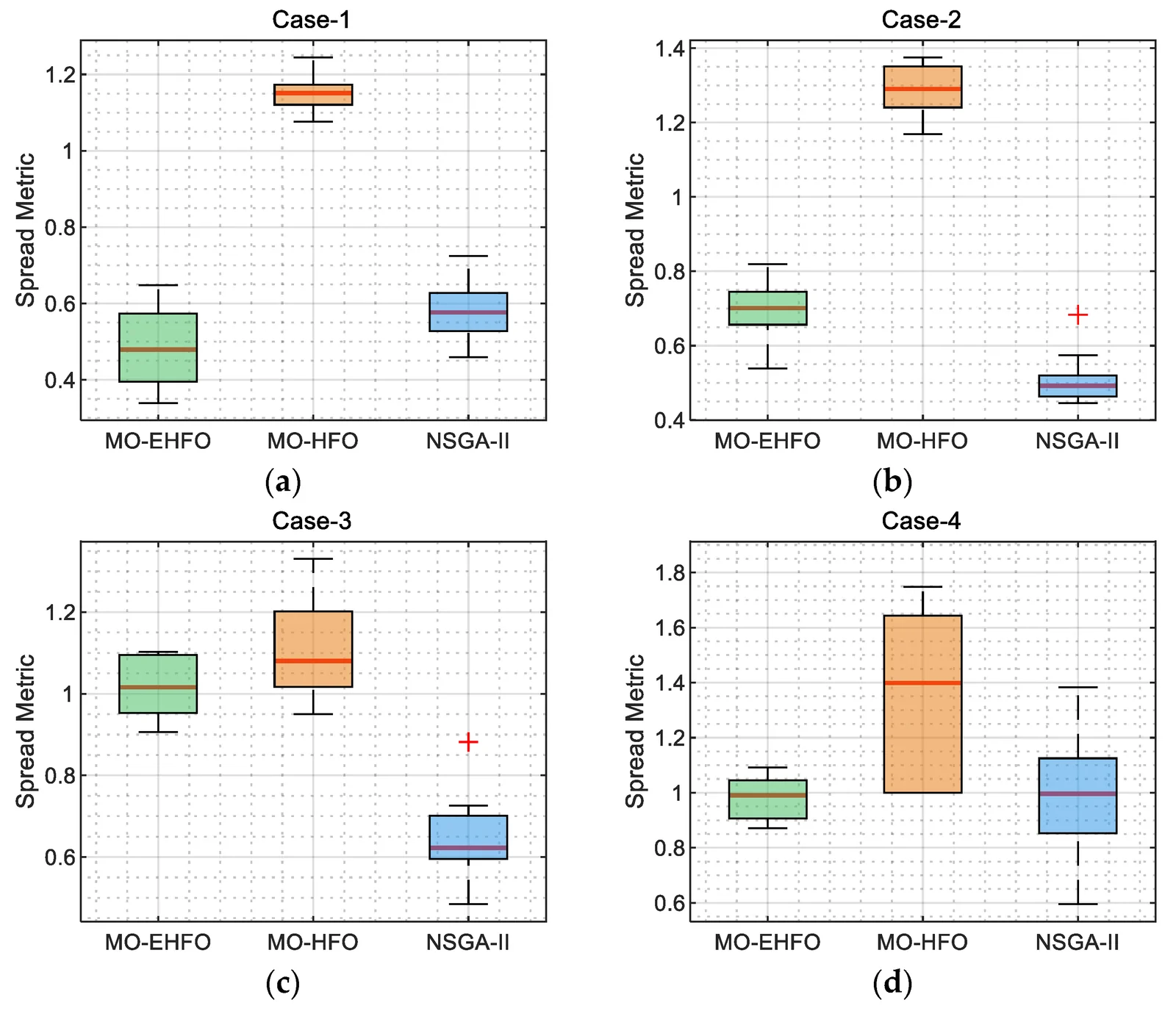
Twenty runs of each algorithm resulting in Δ-box plots: (**a**) cost–emission, (**b**) cost–loss, (**c**) cost–VD, (**d**) cost–L-index. In each box plot, the central red line indicates the median, the lower and upper edges of the box denote the 25th and 75th percentiles, the black whiskers extend to the most extreme data points not considered outliers, and the red ‘+’ markers represent the outliers.

As the Δ-box plots in Figure 6 and the spread columns of Table 11 jointly show, MO-EHFO and NSGA-II maintain a homogeneous distribution on the simpler fronts, whereas MO-HFO is consistently the most poorly spread. For Case-1, MO-EHFO attains the lowest spread (Δ = 0.4865), followed by NSGA-II (0.5815), while MO-HFO is markedly worse (1.1536). For Case-2, NSGA-II yields the most uniform front (Δ = 0.5082), ahead of MO-EHFO (0.6879), with MO-HFO again clustered (1.2815). On the non-convex Case-3 front, NSGA-II is clearly the most uniform (Δ = 0.6514), whereas both honey-formation variants spread less evenly (MO-EHFO 1.014, MO-HFO 1.1122). The most telling result appears on the hardest Case-4 front: the spread of MO-HFO is undefined (NaN) because its archive collapses to fewer than two non-dominated solutions, while MO-EHFO retains the only well-defined, compact box (Δ = 0.9826) and NSGA-II becomes more scattered (1.0011 ± 0.2247). These trends follow directly from the search mechanisms: the scalar HoneyCost of MO-HFO (Equation (46)) biases the swarm toward a single region and degrades diversity, whereas the crowding-distance smart pruning and chaotic initialization of MO-EHFO, together with the explicit crowding operator of NSGA-II, preserve an even coverage.

#### 5.5.2. Hypervolume Indicator (HV)

For a problem in which all objectives are to be minimized, the HV metric measures the volume of the objective-space region that is jointly dominated by the approximation set Ω and bounded by a reference point W formed from the worst objective values. Each solution i∈Ω defines a hyper-rectangle $v_{i}$ whose opposite corner is W; as given in (64), the indicator equals the volume of the union of these hyper-rectangles [59,60]. Here, the symbol ∪ denotes the mathematical set-union operator.

(64) $$HV=\overset{\left|Ω\right|}{\underset{i=1}{⋃}}v_{i}$$

In this study, the objectives were first scaled to the unit interval using the common ideal and nadir points, the reference point was set to $W=\left(1.1,1.1\right)$ in the normalized space, and the raw volume was divided by the reference hyper-rectangle so that HV lies within the interval $\left[0,1\right]$. A larger HV is preferable, since it reflects an approximation that is both closer to the true front and spread over a wider region. The HV column of Table 11 summarizes the mean, standard deviation, minimum, and maximum of the normalized hypervolume obtained for the four scenarios.

**Figure 7:**
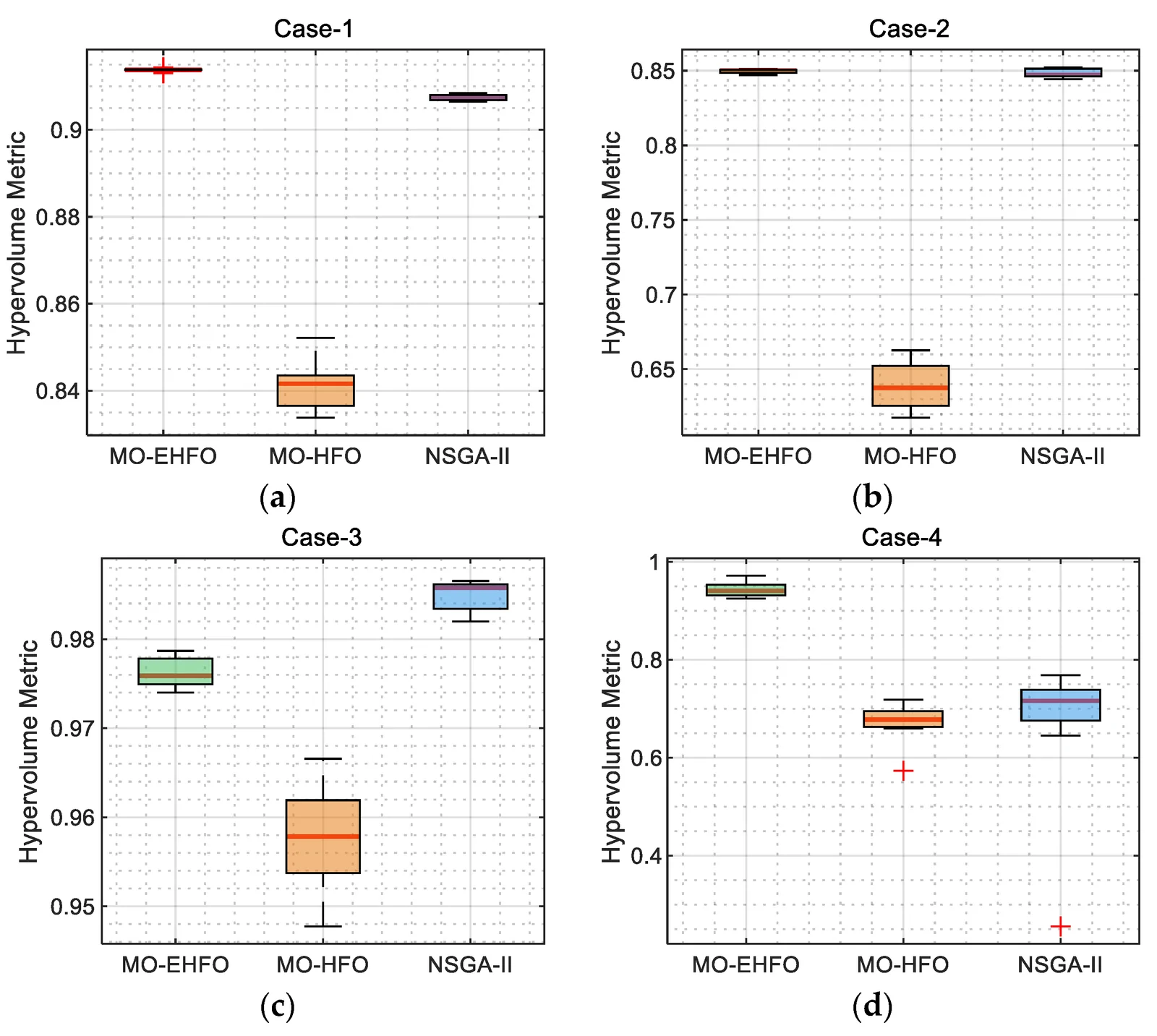
Twenty runs of each algorithm resulting in HV-box plots: (**a**) cost–emission, (**b**) cost–loss, (**c**) cost–VD, (**d**) cost–L-index. In each box plot, the central red line indicates the median, the lower and upper edges of the box denote the 25th and 75th percentiles, the black whiskers extend to the most extreme data points not considered outliers, and the red ‘+’ markers represent the outliers.

The HV-box plots in Figure 7 and the HV columns of Table 11 quantify the overall front quality. On Case-1, MO-EHFO achieves the largest hypervolume (HV = 0.9138) with an exceptionally small dispersion (±0.0004), narrowly ahead of NSGA-II (0.9075) and well above MO-HFO (0.8414). For Case-2, MO-EHFO again leads (0.8495), essentially tied with NSGA-II (0.848), while MO-HFO drops sharply (0.639). On the non-convex Case-3 front, NSGA-II obtains the highest HV (0.9848), closely followed by MO-EHFO (0.9762) and MO-HFO (0.9572), indicating that all methods cover a large dominated volume on this front. The decisive separation occurs on Case-4: MO-EHFO clearly dominates (HV = 0.9438), whereas MO-HFO (0.6727) and especially NSGA-II degrade and destabilize (0.6682 ± 0.1496, with a minimum of only 0.2556). The adaptive exploration-to-exploitation weight (w(t): 1.0→0.4, Equations (51)–(53)) and the archive-guided leader selection of MO-EHFO sustain a large, stable dominated region even as the problem complexity grows, while the purely genetic search of NSGA-II loses reliability on the saturated front.

#### 5.5.3. Inverted Generational Distance (IGD)

The inverted generational distance (IGD) complements the preceding indicators by measuring the average distance from each point of a reference Pareto front $P^{*}$ to its nearest solution in the approximation set A. It ranges from 0 to ∞, where smaller values indicate better convergence together with a more uniform distribution of the obtained front. IGD is defined as in (65).

(65) $$IGD=\frac{1}{\left|P^{*}\right|}\sum_{i=1}^{\left|p*\right|}d(v_{i},A)$$

where $d(v_{i},\;A)$ is the minimum Euclidean distance from the i -th reference point $vᵢ\in P^{*}$ to its closest solution in the approximation set A, and $\left|P^{*}\right|$ is the total number of reference points in $P^{*}$.

Since the true Pareto front is generally unavailable for complex power-system problems, $P^{*}$ was constructed as the non-dominated subset of the union of all solutions produced by the three optimizers over the 20 runs of each case, thereby providing a common, algorithm-independent benchmark. Because IGD simultaneously penalizes poor convergence and incomplete coverage, a smaller value indicates an approximation that is both accurate and well-distributed. Table 11 summarizes the IGD statistics for the four bi-objective cases.

**Figure 8:**
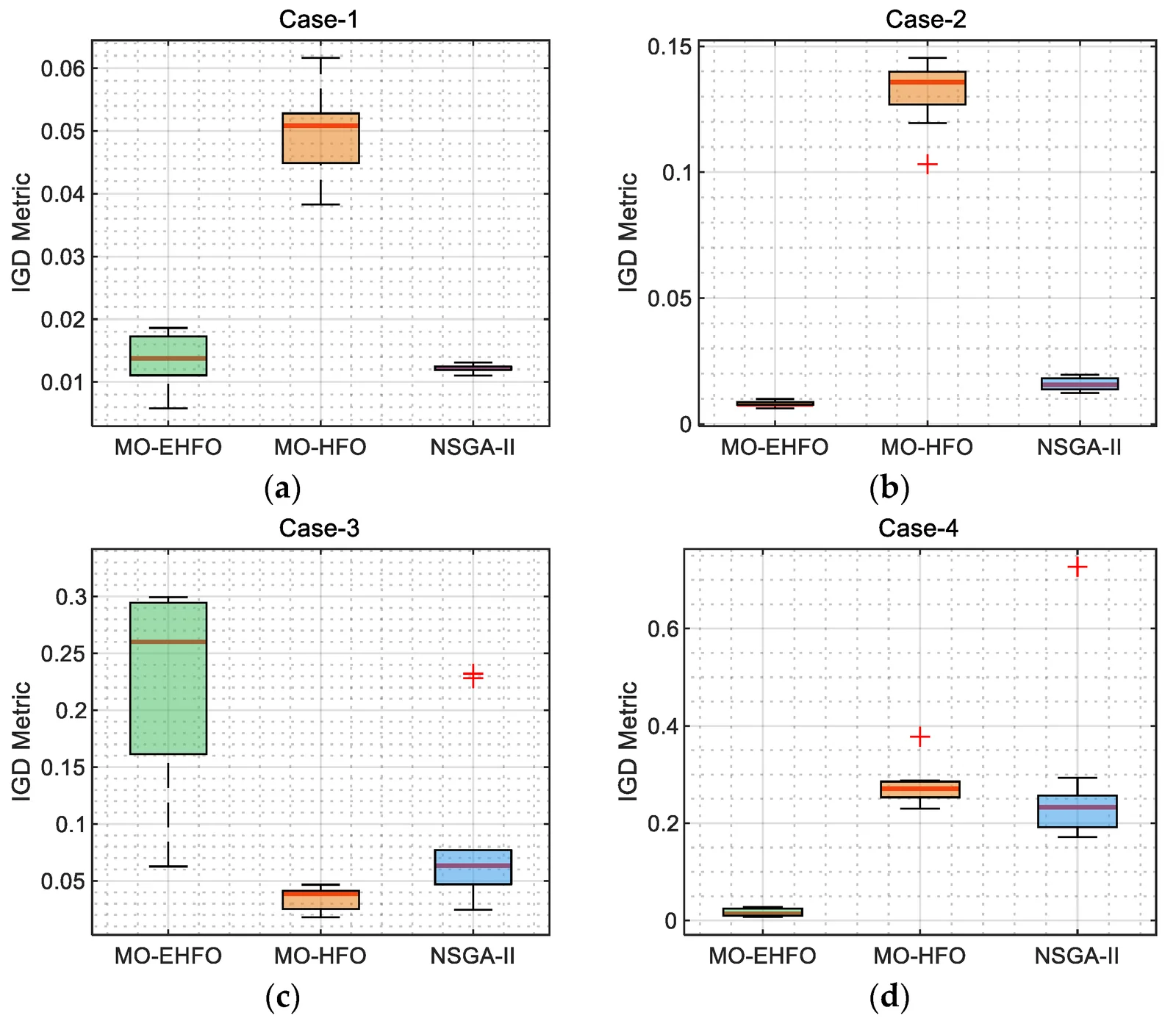
Twenty runs of each algorithm resulting in IGD-box plots: (**a**) cost–emission, (**b**) cost–loss, (**c**) cost–VD, (**d**) cost–L-index. In each box plot, the central red line indicates the median, the lower and upper edges of the box denote the 25th and 75th percentiles, the black whiskers extend to the most extreme data points not considered outliers, and the red ‘+’ markers represent the outliers.

The IGD-box plots in Figure 8 and the IGD columns of Table 11 assess convergence and coverage relative to the reference front P*. MO-EHFO and NSGA-II converge tightly on the simpler fronts: for Case-1 NSGA-II records the lowest IGD (0.0122), marginally ahead of MO-EHFO (0.0133), with MO-HFO worse (0.0498); for Case-2 MO-EHFO attains the best convergence (0.0081), ahead of NSGA-II (0.0157) and far ahead of MO-HFO (0.1314). The non-convex Case-3 front reverses this pattern: MO-HFO unexpectedly yields the lowest IGD (0.0345), whereas MO-EHFO records the highest and least stable value (0.2197 ± 0.0901). This reflects a convergence–coverage trade-off, since the chaotic attraction of MO-EHFO drives the swarm into the low-cost knee of the discontinuous voltage-deviation front, enlarging the dominated volume (high HV) while leaving parts of the pooled reference front under-represented. On the hardest Case-4 front, MO-EHFO recovers a decisive advantage (IGD = 0.0156), while both MO-HFO (0.2755) and NSGA-II (0.2748 ± 0.1632) converge poorly and inconsistently.

Considered jointly, the three indicators yield a balanced assessment: HV rewards the overall quality of the front, IGD emphasizes convergence and coverage with respect to the reference set, and Δ focuses on the uniformity of the distribution. An optimizer is deemed superior for a given case when it simultaneously attains the largest HV together with the smallest IGD and Δ. The values reported in Table 11 are obtained directly from the box-plot statistics computed for each indicator, allowing the relative ranking of MO-EHFO, MO-HFO, and NSGA-II to be read consistently across all four scenarios.

### 5.6. General Discussion of the Findings

This section presents a comparative discussion of the performance of the proposed method relative to existing methods reported in the literature. The objective function values obtained using the algorithms in this study were evaluated with respect to their respective algorithmic mechanisms, and the reasons for the superior performance of the proposed approach were explained. Except for MO-HFO Case 1.a, low production costs were achieved in all other cases, but environmental and technical objectives were compromised. Because MO-HFO does not include the advanced archive management and diversity preservation mechanisms introduced by MO-EHFO, it tends to converge to economically attractive regions of the search space. This also explains why, due to POZ-induced constraints, MO-HFO fails to converge in fragmented regions of the search space during optimization. As a result, it leads to cost improvements rather than a truly Pareto-optimal equilibrium. NSGA-II has demonstrated poor performance in complex and conflicting OPF search spaces with numerous local optima and nonlinear operational constraints, such as Cases 2, 3, 4, and especially 8, characterized by slower convergence and weaker exploitation capabilities. In cases where it yielded cost-advantageous results compared to MO-EHFO, it inevitably compromised technical and environmental objectives.

Both NSGA-II and MO-HFO, as well as studies reported in the literature, demonstrate performance that improves a single objective at the expense of significantly worsening one or more other objectives in multi-objective optimization. MO-EHFO’s adaptive search and archive-driven mechanisms continue to ensure an efficient exploration process and convergence toward Pareto-optimal solutions with good diversity. This demonstrates that the proposed search mechanisms not only accelerate convergence but also improve the quality and diversity of the resulting Pareto front, thereby identifying physically meaningful operating points that satisfy all safety and operational constraints. MO-EHFO not only demonstrated consistency across all examined two-, three-, and four-objective OPF case studies but also maintained this consistency across different optimization scenarios while satisfying all operational constraints.

## 6. Conclusions

This study makes a significant contribution to the literature on multi-objective optimization by proposing the MO-EHFO algorithm. Given that preliminary test results for the MO-HFO algorithm indicated weak performance in convergence to the Pareto front and diversity, the need to propose the MO-EHFO algorithm has emerged. To surmount the limitations of MO-HFO, this robust enhancement integrates pure Pareto-dominance-based decision mechanisms. The deployment of the algorithm on the modified IEEE 30-bus test system consistently enabled MO-EHFO to attain the reference Pareto front. Throughout the evaluations, all complex and nonlinear power system security and operational constraints were transparently defined and strictly enforced. To further evaluate the performance of the mentioned algorithms, we conducted a metric-based assessment across 20 independent runs, which corroborated these findings. Across the four bi-objective scenarios, MO-EHFO consistently provides the best balance of convergence (IGD), front quality (HV), and diversity (Δ). The numerical results further confirm the effectiveness of the proposed approach. It achieved the highest hypervolume in Case 1 (0.9138) and demonstrated its greatest advantage in the most challenging Case 4, where it obtained HV and IGD of 0.9438 and 0.0156, respectively, while NSGA-II achieved only 0.6682 and 0.2748. In the four-objective Case 8, MO-EHFO reaches 920.7131 $/h, 0.2459 ton/h, 3.2316 MW, and 0.2323 p.u., corresponding to reductions of 2.3% in cost, 3.9% in emissions, 28.8% in active power loss, and 61.37% in voltage deviation relative to the best previously reported literature results.

This improvement stems directly from the enhancements introduced in MO-EHFO pure Pareto dominance with crowding distance, logistic-map chaotic initialization, the adaptive exploration-to-exploitation weight, and the smart archive with personal Pareto memories. The seasonal scenario-based analyses further demonstrated the robustness and stability of MO-EHFO under stochastic renewable generation. The seasonal validation revealed that the proposed algorithm preserved the same ordering of the best and worst compromise scenarios under both unconstrained and POZ-constrained operating conditions, while maintaining physical feasibility and operational reliability, thereby providing strong evidence of its robustness against changes in renewable generation patterns and network operating constraints. It is hypothesized that future applications of this algorithm could extend to larger-scale, real-world power grids and smart grid environments dealing with highly dynamic loads.

## Figures and Tables

**Table 1:** RES system parameters of wind farms, PV plant, and hydro plant considered for IEEE 30-bus test system [31].

Turbine and PDF Parameters	Wind	PV	PV + Hydro
	Bus No 5	Bus No 11	Bus No 13
$v_{in}$ (m/s)	3		
$v_{r}$ (m/s)	16		
$v_{out}$ (m/s)	25		
$P_{r}$ [MW]	75	50	45 + 5
c–Weibull	9		
k–Weibull	2		
$G_{s}$ [W/m^2^]			483
$\mu_{S}$ and σ		5.2 and 0.6	5 and 0.6
$\mu_{H}$ and β			15 and 1.2

**Table 2:** Modified IEEE 30-bus test system.

Items	Quantity	Description	Range
Buses	30	[46]	
Branches	41	[46]	
Thermal generators	3	Buses: 1 (Slack), 2, 8	
Wind generator	1	Buses: 5	
Solar PV unit	1	Bus: 11	
Solar PV + Hydro	1	Bus: 13	
Control variables	11	[*P*_G2_, *P*_G5_, *P*_G8_, *P*_G11_, *P*_G13_] and [*V*_G1_, *V*_G2_, *V*_G5_, *V*_G8_, *V*_G11_, *V*_G13_]	
$P_{D}$ + j$Q_{D}$		283.4 MW + j126.2 MVAr	
Generators Voltage	6		[0.95–1.1]
Load Buses Voltage	24		[0.95–1.05]

**Table 3:** Summary of case studies.

	Case Number	$F_{CT}$	$F_{E}$	$F_{PL}$	$F_{VD}$	$F_{VSI}$
Bi-Objective	Case-1	✔	✔			
	Case-1a (Poz)	✔	✔			
	Case-2	✔		✔		
	Case-3	✔			✔	
	Case-4	✔				✔
Triple-Objective	Case-5	✔	✔	✔		
	Case-6	✔	✔		✔	
	Case-7	✔	✔			✔
Quad-Objective	Case-8	✔	✔	✔	✔	

✔ indicates that the corresponding objective/constraint is considered in that case study.

**Table 4:** Simulation results of case studies 1–1a on IEEE 30-bus test system.

		Case-1			Case-1a (POZ)				
Items	Range	NSGA-II	MO-HFO	MO-EHFO	MOEA/D-SF [25]	SMODE-SF [25]	NSGA-II	MO-HFO	MO-EHFO
*P*_G1T_	[30 140]	92.7166	95.9359	85.3616	117.118	111.91	91.5599	79.2275	83.7365
*P*_G2T_	[20 80] *	54.5221	52.4630	50.9104	65	65	52.3642	65.4410	53.5092
*P*_G5W_	[0 75]	54.3548	75.0000	60.5397	55.447	54.058	61.8450	57.6900	58.6452
*P*_G8T_	[10 35]	30.4514	15.5805	34.3121	18.403	23.555	31.4526	30.6490	35.0000
*P*_G11_	[0 50]	49.6988	43.7273	49.3021	17.649	18.436	37.8871	39.9125	49.3496
*P*_13GPV+Hydro_	[0 50]	5.9405	5.1671	6.5037	15.326	15.755	12.4026	14.4752	6.8408
*V*_1_	[0.95 1.1]	1.0866	1.0033	1.0455	1.076	1.0761	1.0049	1.0560	1.0214
*V*_2_	[0.95 1.1]	1.0224	1.0426	1.0192	1.0648	1.0662	0.9937	1.0410	0.9822
*V*_5_	[0.95 1.1]	1.0364	1.0086	1.0270	1.0444	1.0362	0.9744	0.9975	1.0016
*V*_8_	[0.95 1.1]	1.0625	0.9751	1.0481	1.0402	1.0362	0.9911	1.0067	1.0337
*V*_11_	[0.95 1.1]	1.0233	0.9911	1.0471	1.0878	1.0778	1.0684	1.0363	1.0544
*V*_13_	[0.95 1.1]	0.9764	1.0430	1.0395	1.0602	1.0432	1.0444	1.0152	1.0279
*Q*_1_	[−50 140]	41.7086	−18.6197	−5.9340	2.128	2.788	−2.0218	22.2392	−1.2309
*Q*_2_	[−20 60]	5.9817	34.6147	15.2261	21.41	34.504	−0.2430	28.2037	4.2065
*Q*_5_	[−30 35]	22.7072	25.5310	26.9430	37.727	36.169	15.5347	5.2590	27.5492
*Q*_8_	[−15 40]	31.6301	25.1203	37.7816	27.102	20.376	35.9736	22.8921	33.4767
*Q*_11_	[−20 25]	−2.3434	−0.0829	4.9623	24.911	23.41	20.9554	6.0909	14.4346
*Q*_13_	[−20 25]	−18.0644	17.1303	−1.5217	20.328	15.62	10.6509	−6.1930	1.7711
$Q_{Loss}$ (MVAr))		−0.2128	−0.1921	−0.2544	-	-	−0.2205	−0.2441	−0.2269
$F_{CW}$ ($/h)		890.3921	893.2257	**901.7422**	919.040	927.049	900.9256	920.7629	**904.3035**
$F_{E}$ (ton/h)		0.2561	0.2603	**0.2506**	0.6221	0.4721	0.2549	0.2497	**0.2498**
$F_{PL}$ (MW)		4.2841	4.4739	3.5295	5.5429	5.3148	4.1114	3.9951	3.6812
$F_{VD\;}$ (p.u)		0.4270	0.4320	0.5616	0.4530	0.4215	0.3496	0.2582	0.2530
$F_{VSI}$		0.1361	0.1490	0.1357	3.50	3.296	0.1441	0.1408	0.1417

Bold values indicate the best result. * denotes the generator subject to the prohibited operating zones (POZ) constraint.

**Table 5:** Simulation results of case studies 2–4 on IEEE 30-bus test system.

		Case-2			Case-3			Case-4		
Items	Range	NSGA-II	MO-HFO	MO-EHFO	NSGA-II	MO-HFO	MO-EHFO	NSGA-II	MO-HFO	MO-EHFO
*P*_G1T_	[30 140]	89.9354	92.2889	91.0303	139.9203	139.7750	139.9056	138.2361	139.2000	138.9549
*P*_G2T_	[20 80]	30.0650	47.2616	30.4563	44.2682	42.0389	42.6882	35.9737	43.2230	42.2293
*P*_G5W_	[0 75]	74.1770	75.0000	74.5173	47.6664	49.4485	49.3279	49.7933	48.7081	49.0652
*P*_G8T_	[10 35]	32.7972	15.7020	34.4305	10.0809	10.0000	10.0880	17.2682	10.1869	11.0427
*P*_G11_	[0 50]	48.1790	48.7917	48.8352	41.7491	41.7376	41.7051	41.7659	42.0019	42.0397
*P*_13GPV+Hydro_	[0 50]	11.3917	8.6041	7.2963	6.2856	6.8114	6.1946	6.1099	6.1304	6.1426
*V*_1_	[0.95 1.1]	1.0445	1.0487	1.0519	1.0424	1.0718	1.0388	1.0806	1.0831	1.0785
*V*_2_	[0.95 1.1]	0.9804	1.0136	1.0130	1.0031	1.0305	1.0380	1.0911	1.0720	1.0846
*V*_5_	[0.95 1.1]	1.0302	1.0180	1.0256	1.0339	1.0175	1.0094	1.0630	1.0442	1.0536
*V*_8_	[0.95 1.1]	1.0618	1.0005	1.0507	1.0400	1.0444	1.0147	1.0473	1.0444	1.0770
*V*_11_	[0.95 1.1]	1.0677	0.9997	1.0578	1.0378	1.0041	1.0084	1.0380	1.0409	1.0374
*V*_13_	[0.95 1.1]	1.0512	0.9765	1.0420	1.0120	1.0080	1.0143	1.0362	1.0373	1.0368
*Q*_1_	[−50 140]	7.5294	41.2426	−1.4456	11.8081	22.7420	−6.1290	−7.1676	−2.4192	−18.7793
*Q*_2_	[−20 60]	−0.4394	−0.7714	18.7348	9.6032	26.3311	28.3187	33.8851	36.8173	47.9763
*Q*_5_	[−30 35]	22.2076	26.7275	17.2690	28.3436	19.6351	29.4323	29.8268	21.5370	28.6452
*Q*_8_	[−15 40]	31.5933	25.8382	36.1553	33.3091	32.7279	39.6405	39.9462	39.5513	39.9076
*Q*_11_	[−20 25]	12.0895	2.0067	7.2834	9.2472	−4.9919	−0.6068	−4.2864	−2.8585	−4.5298
*Q*_13_	[−20 25]	2.4705	−11.4668	−2.4420	−3.6117	−8.8506	−2.1480	−9.8145	−9.1596	−9.7406
$Q_{Loss}$(MVAr))		−0.2745	−0.1932	−0.2734	−0.1420	−0.1531	−0.1439	−0.2051	−0.1943	−0.1942
$F_{CW}$($/h)		910.3383	899.6483	**904.9426**	850.1572	850.2454	**850.1972**	850.9161	848.6354	**849.1218**
$F_{E}$(ton/h)		0.2553	0.2573	0.2559	0.3120	0.3119	0.3120	0.3086	0.3109	0.3104
$F_{PL}$(MW)		3.1453	4.2484	**3.1658**	6.5704	6.4115	6.5094	5.7470	6.0503	6.0744
$F_{VD\;}$(p.u)		0.6146	0.4234	0.6585	0.2144	0.2535	**0.1977**	0.8813	0.8616	0.8869
$F_{VSI}$		0.1359	0.1474	0.1346	0.1430	0.1401	0.1429	0.1312	0.1316	**0.1312**

Bold values indicate the best result.

**Table 6:** Simulation results of case studies 5–7 on IEEE 30-bus test system.

		Case-5			Case-6			Case-7		
Items	Range	NSGA-II	MO-HFO	MO-EHFO	NSGA-II	MO-HFO	MO-EHFO	NSGA-II	MO-HFO	MO-EHFO
*P*_G1T_	[30 140]	77.5872	93.9316	74.3668	95.4973	104.6415	89.2785	112.9433	114.9602	83.1789
*P*_G2T_	[20 80]	48.0533	51.4999	46.9650	55.9177	55.7092	52.1050	48.3220	44.9080	47.6564
*P*_G5W_	[0 75]	71.5194	57.0272	74.4058	58.4664	54.8831	64.2274	49.3043	47.1286	70.0313
*P*_G8PV_	[10 35]	32.1176	23.9348	34.3562	23.0619	17.1896	29.3185	24.0352	24.3694	30.4899
*P*_G11PV+Hydro_	[0 50]	47.1572	42.1559	48.8741	45.3149	47.5078	45.4902	44.1005	37.9685	47.8424
*P*_G13T_	[0 50]	10.1264	19.0188	7.3582	9.4304	8.2596	6.8312	9.4980	19.0521	7.5272
*V*_1_	[0.95 1.1]	1.0528	1.0552	1.0400	1.0479	1.0285	1.0463	1.0817	1.0503	1.0738
*V*_2_	[0.95 1.1]	1.0259	1.0180	1.0014	1.0282	0.9872	1.0356	1.0367	1.0041	1.0833
*V*_5_	[0.95 1.1]	1.0167	1.0404	1.0138	1.0085	1.0275	1.0058	1.0522	1.0036	1.0669
*V*_8_	[0.95 1.1]	1.0141	1.0068	1.0380	1.0144	1.0045	1.0435	1.0678	1.0337	1.0487
*V*_11_	[0.95 1.1]	1.0609	1.0537	1.0610	1.0581	1.0567	1.0108	1.0452	1.0791	1.0379
*V*_13_	[0.95 1.1]	1.0359	1.0167	1.0451	1.0071	1.0313	1.0129	1.0283	1.0497	1.0257
*Q*_1_	[−50 140]	21.7571	20.5311	1.6812	22.6523	1.0698	7.4001	12.9244	8.8763	−12.7285
*Q*_2_	[−20 60]	3.0576	9.4576	12.7533	−4.4441	14.6729	26.7852	23.0701	13.1568	43.2866
*Q*_5_	[−30 35]	18.8271	28.4161	16.9495	20.8353	24.0636	13.3729	24.7235	15.0325	25.4605
*Q*_8_	[−15 40]	22.3355	16.7013	30.8911	38.1429	24.5533	38.6459	29.7791	25.1040	36.4039
*Q*_11_	[−20 25]	11.6376	11.0489	11.2786	13.2294	15.9367	−1.4022	0.4342	16.4194	−3.5079
*Q*_13_	[−20 25]	−1.6509	−7.3942	2.0875	−9.5181	3.3248	−4.9650	−11.0763	1.6387	−13.3594
$Q_{Loss}$ (MVAr))		−0.2694	−0.2414	−0.2726	−0.2200	−0.1928	−0.2306	−0.2305	−0.2267	−0.2734
$F_{CW}$ ($/h)		920.4789	904.8058	**923.7084**	887.6461	870.9783	**897.3254**	859.6125	874.0991	**909.2190**
$F_{E}$ (ton/h)		0.2462	0.2574	**0.2445**	0.2591	0.2677	**0.2535**	0.2743	0.2764	**0.2494**
$F_{PL}$ (MW)		3.1610	4.1682	**2.9262**	4.2886	4.7909	3.8509	4.8032	4.9867	3.3261
$F_{VD\;}$ (p.u)		0.4779	0.3326	0.5157	0.2831	0.2723	**0.2196**	0.7511	0.5727	0.8451
$F_{VSI}$		0.1377	0.1398	0.1371	0.1397	0.1428	0.1409	0.1330	0.1370	**0.1313**

Bold values indicate the best result.

**Table 7:** Simulation results of case study 8 on IEEE 30-bus test system.

		Case-8					Case-8		
Items	Range	NSGA-II	MO-HFO	MO-EHFO	Items	Range	NSGA-II	MO-HFO	MO-EHFO
*P*_G1T_	[30 140]	68.2500	93.1532	76.9200	*Q*_1_	[−50 140]	−6.9428	−17.6762	3.8023
*P*_G2T_	[20 80]	54.0603	46.4996	50.6729	*Q*_2_	[−20 60]	12.1220	23.9207	20.3818
*P*_G5W_	[0 75]	61.5430	75.0000	69.8187	*Q*_5_	[−30 35]	27.4272	28.4831	18.6906
*P*_G8PV_	[10 35]	29.2915	24.6200	32.9469	*Q*_8_	[−15 40]	29.5325	36.1950	39.3328
*P*_G11PV+Hydro_	[0 50]	45.4811	43.8490	45.9602	*Q*_11_	[−20 25]	16.2370	18.3343	−0.1756
*P*_G13T_	[0 50]	27.8914	4.1712	10.3127	*Q*_13_	[−20 25]	−2.1362	−8.9726	−4.7018
*V*_1_	[0.95 1.1]	1.0235	1.0246	1.0403	$Q_{Loss}$ (MVAr))		−0.2666	−0.2262	−0.2557
*V*_2_	[0.95 1.1]	0.9989	1.0130	1.0218	$F_{CW}$ ($/h)		965.7267	901.0483	**920.7131**
*V*_5_	[0.95 1.1]	1.0584	1.0165	1.0115	$F_{E}$ (ton/h)		0.2427	0.2565	**0.2459**
*V*_8_	[0.95 1.1]	1.0069	1.0394	1.0352	$F_{PL}$ (MW)		3.1174	3.8930	3.2316
*V*_11_	[0.95 1.1]	1.0646	1.0696	1.0158	$F_{VD\;}$ (p.u)		0.3132	0.2384	0.2323
*V*_13_	[0.95 1.1]	1.0262	1.0031	1.0151	$F_{VSI}$		0.1406	0.1407	0.1405

Bold values indicate the best result.

**Table 9:** Stochastic scenarios for test system with RESs.

Scenarios No.	Scenario Probability	Wind Speed $v\left(m/s\right)$	Irradiance $G(W/m^{2})$	Flow Rate $Q(m^{3}/s)$	Case 1		Case 1a	
					Cost	Emis.	Cost	Emis.
1	0.0110	5.9580	596.3083	14.0100	901.3090	0.2670	892.4888	0.2715
2	0.0003	13.8493	1082.2330	16.0344	1291.9653	0.2385	1286.5582	0.2388
3	0.0008	7.1567	1021.4347	14.4500	1088.4342	0.2483	1092.1217	0.2462
4	0.0003	3.4128	1263.2603	12.6728	1127.5808	0.2557	1128.1238	0.2553
5	0.0278	6.2007	497.8796	14.1059	872.1972	0.2742	877.0852	0.2720
6	0.0001	12.3344	2325.9957	15.7564	1247.5994	0.2387	1249.5088	0.2385
7	0.0715	7.2310	334.7051	14.4748	855.9253	0.2810	855.9883	0.2810
8	**0.3800**	8.6482	142.9109	14.9043	878.7477	0.2953	878.6246	0.2957
9	0.0035	5.4320	700.0901	13.7882	936.9530	0.2625	932.8140	0.2645
10	0.0003	12.6932	1333.6194	15.8252	1258.1746	0.2388	1260.8259	0.2387
11	0.0043	10.9106	744.2023	15.4620	1025.9070	0.2495	1028.7485	0.2477
12	0.0015	10.2510	815.8631	15.3124	1041.4888	0.2481	1044.7819	0.2461
13	0.0361	6.7736	395.1263	14.3179	854.1920	0.2795	854.2798	0.2795
14	0.1434	6.3241	281.4910	14.1531	851.9949	0.2947	853.9265	0.2901
15	0.0016	8.2886	908.1491	14.8024	1045.4494	0.2498	1048.6033	0.2478
16	0.0009	2.0272	862.8192	11.4226	1017.0762	0.2618	1012.9752	0.2637
17	0.0655	4.4708	56.1808	13.3208	894.5339	0.3365	894.5345	0.3365
18	0.0039	7.5049	654.3005	14.5640	**930.3999**	**0.2596**	**926.3887**	**0.2616**
19	0.0004	11.5691	1195.4393	15.6027	1224.6011	0.2391	1228.8303	0.2383
20	0.0004	3.6690	1142.4111	12.8464	1126.2833	0.2549	1127.3302	0.2542
21	0.1459	6.0786	101.2802	14.0581	874.6578	0.3157	874.6830	0.3157
22	0.0785	8.1205	212.7892	14.7532	866.0673	0.2889	866.2761	0.2885
23	0.0220	8.1805	439.0700	14.7709	878.1294	0.2700	886.8003	0.2664
24	0.0001	9.8526	1881.3309	15.2172	1180.3775	0.2415	1179.0140	0.2426
25	0.0003	6.0190	1581.7716	14.0345	1127.3611	0.2480	1130.9300	0.2459

Bold values indicate the best result.

**Table 10:** Control parameters of the compared algorithms.

Parameter	MO-EHFO (Proposed)	MO-HFO (Baseline)	NSGA-II
Population size, $N_{pop}$	50	50	50
External Pareto archive, $N_{A}$	200	200	(elitist population)
Per-bee personal archive	5	-	-
Mixing-period factor, MF	8	8	-
Recombination	Honey-form mixing, 50% crossover	Honey-form mixing, 50% crossover	SBX, $p_{c}$ = 0.90, $\eta_{c}$ = 20
Max. function evaluations	25,000	25,000	25,000
Independent runs	20	20	20

**Table 11:** Comparative analysis of the spread (Δ), HV, and IGD indicator of multi-objective algorithms across 20 runs, including mean, ±standard deviation, minimum, and maximum values.

Case Study		Spread (Δ) (Smaller Is Better)				HV (Larger Is Better)				IGD (Smaller Is Better)			
		Avg.	Std.	Min.	Max.	Avg.	Std.	Min.	Max.	Avg.	Std.	Min.	Max.
**Case-1**	**MO-EHFO**	**0.4865**	0.1059	**0.3395**	**0.6484**	**0.9138**	**0.0004**	**0.913**	**0.9144**	0.0133	0.0047	**0.0058**	0.0186
	MO-HFO	1.1536	**0.0464**	1.076	1.2442	0.8414	0.0059	0.8338	0.8521	0.0498	0.0063	0.0383	0.0617
	NSGA-II	0.5815	0.0811	0.4595	0.7242	0.9075	0.0007	0.9065	0.9084	**0.0122**	**0.0005**	0.0111	**0.0131**
**Case-2**	**MO-EHFO**	0.6879	0.0872	0.5385	0.8193	**0.8495**	**0.0014**	**0.8471**	0.8511	**0.0081**	**0.0011**	**0.0062**	**0.0099**
	MO-HFO	1.2815	**0.0707**	1.1686	1.375	0.639	0.0147	0.6175	0.6627	0.1314	0.0124	0.1032	0.1454
	NSGA-II	**0.5082**	0.0724	**0.4459**	**0.6828**	0.848	0.003	0.8442	**0.8523**	0.0157	0.0025	0.0124	0.0195
**Case-3**	MO-EHFO	1.014	**0.0742**	0.9059	1.1026	0.9762	**0.0017**	0.974	0.9787	0.2197	0.0901	0.0627	0.2995
	MO-HFO	1.1122	0.1335	0.9501	1.3307	0.9572	0.0056	0.9478	0.9666	**0.0345**	**0.0099**	**0.018**	**0.0467**
	**NSGA-II**	**0.6514**	0.107	**0.4843**	**0.8819**	**0.9848**	0.0018	**0.982**	**0.9865**	0.0902	0.0755	0.0247	0.2321
**Case-4**	**MO-EHFO**	**0.9826**	**0.0784**	0.8713	**1.0923**	**0.9438**	**0.0147**	**0.9251**	**0.9714**	**0.0156**	**0.0075**	**0.0074**	**0.0274**
	MO-HFO	NaN	NaN	1	1.7481	0.6727	0.04	0.5731	0.718	0.2755	0.0407	0.23	0.3779
	NSGA-II	1.0011	0.2247	**0.5948**	1.3826	0.6682	0.1496	0.2556	0.7687	0.2748	0.1632	0.1713	0.7272

Bold values indicate the best result.

## Data Availability

The datasets used and analyzed in this study were obtained from the website https://icseg.iti.illinois.edu/ieee-30-bus-system (accessed on 5 January 2026).
